# Ternary Logic with Stateful Neural Networks Using a Bilayered TaO*_X_*‐Based Memristor Exhibiting Ternary States

**DOI:** 10.1002/advs.202104107

**Published:** 2021-12-16

**Authors:** Young Seok Kim, Jangho An, Jae Bum Jeon, Myeong Won Son, Seoil Son, Woojoon Park, Younghyun Lee, Juseong Park, Geun Young Kim, Gwangmin Kim, Hanchan Song, Kyung Min Kim

**Affiliations:** ^1^ Department of Materials Science and Engineering KAIST 291 Daehak‐ro, Yuseong‐gu Daejeon 34141 Republic of Korea

**Keywords:** in‐memory computing, memristors, neural networks, stateful logic, ternary logic

## Abstract

A memristive stateful neural network allowing complete Boolean in‐memory computing attracts high interest in future electronics. Various Boolean logic gates and functions demonstrated so far confirm their practical potential as an emerging computing device. However, spatio‐temporal efficiency of the stateful logic is still too limited to replace conventional computing technologies. This study proposes a ternary‐state memristor device (simply a ternary memristor) for application to ternary stateful logic. The ternary‐state implementable memristor device is developed with bilayered tantalum oxide by precisely controlling the oxygen content in each oxide layer. The device can operate 157 ternary logic gates in one operational clock, which allows an experimental demonstration of a functionally complete three‐valued Łukasiewicz logic system. An optimized logic cascading strategy with possible ternary gates is ≈20% more efficient than conventional binary stateful logic, suggesting it can be beneficial for higher performance in‐memory computing.

## Graphical abstract

A ternary state memristor is reported that exhibits two abrupt switching functions, by stacking two layers of tantalum oxide. Ternary logic gates including a functionally complete system and a ternary full adder are experimentally demonstrated using stateful neural networks, which is complete in‐memory computing. The ternary full adder is proven to be more efficient than the binary one.

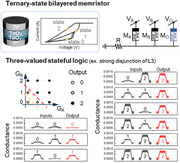

## 1 Introduction

Conventional computing systems suffer from the von Neumann bottleneck problem, which has resulted from the drastic increase in data transfer rates between memory and logic units. To address this, in‐memory computing (or near‐memory computing) techniques that do not require a data bus have received significant interest in efforts to develop next‐generation computing.^[^ ^1^, ^2^, ^3^, ^4^ ^]^ Among the various solutions that have been proposed,^[^ ^5^, ^6^, ^7^ ^]^ memristive stateful logic is considered quite promising.^[^ ^8^, ^9^, ^10^, ^11^, ^12^, ^13^, ^14^, ^15^, ^16^ ^]^ It utilizes the resistance values stored in the memristor cells as logical variables and performs logic operations inside the memristor array by applying appropriate operating voltages to selected cells. As a result, the logical output can be stored in a defined location in the array. In this way, no data transfer is needed for logic operations, which allows complete in‐memory computing most straightforwardly compared to all other technologies.

Multivalued computing is another interesting approach in next‐generation computing. It utilizes more than two logical states for computing, compared with conventional Boolean logic (BL), which utilizes binary states, True (1) and False (0).^[^ ^17^ ^]^ Multivalued computing can increase computational efficiency by reducing the size of data. Jan Łukasiewicz proposed the first modern form of multivalued (or many‐valued) logic in 1920.^[^ ^18^ ^]^ He added a third state between the two BL states (i.e., True and False) and theoretically suggested some ternary gates. Following his theory, we also use a ternary numeral system to express the ternary states: 0 (False in BL), 1, and 2 (True in BL).

The initial three‐valued Łukasiewicz logic (Ł3) consisted of inversion (INV) and implication (IMP) gates. However, the logic was not functionally complete yet; their cascading could not reproduce all ternary gates. Later, it was found that introducing a so‐called “T‐function” (*T*()), which always produces the state 1 output regardless of the inputs, to Ł3 can realize the functionally complete algebra, which can be given as < *E*, IMP, INV, *T*(), 0, 2>, where *E* = {0, 1, 2}.^[^ ^19^, ^20^, ^21^ ^]^ This confirmed that ternary logic could be used in computing.

Since then, studies have proposed various devices to implement ternary logic systems.^[^ ^22^, ^23^, ^24^, ^25^, ^26^ ^]^ Among these studies, some have dealt with the ternary logic using a memristive crossbar array.^[^ ^27^, ^28^, ^29^, ^30^, ^31^, ^32^, ^33^ ^]^ This previous memristive ternary logic was achieved in a nonstateful logic manner; the form of inputs were voltages, not the resistances of the cells. In more detail, the multivalued states were provided by the voltage amplitudes, which can be produced at the periphery circuits. Therefore, these methods can be considered to belong to the near‐memory computing regime.^[^ ^34^ ^]^

Ternary memristive in‐memory computing is possible only if the memristive cell supports ternary states, which has not been demonstrated yet. Even though some studies have proposed multilevel states in some memristive systems, a more detailed strategy is needed to realize memristive ternary logic.

In this study, we propose a reliable ternary‐state memristor system which is capable of achieving ternary logic in a stateful logic manner. The device is composed of a bilayer of tantalum oxides, and it exhibits three distinct and stable resistance states, which permit the ternary states to be realized with resistance. In our investigation based on a stateful neural network theory, 157 ternary gates are theoretically executable in one voltage clock with the device. Afterward, we experimentally demonstrated functionally complete three‐valued Łukasiewicz logic gates and the ternary full adder operations. We examined that the ternary full adder was about 20% more efficient than a state‐of‐the‐art binary full adder.

## 2 Ternary State Memristor Device

Ternary stateful logic requires a memristor with three discrete states (namely, a high resistance state (HRS), an intermediate resistance state (IRS), and a low resistance state (LRS)). Also, the transition between the states should be sharp to ensure a clear distinction between the states. To meet the requirements, we theoretically devised a serial configuration of two distinct memristive components (M1 and M2). We designated the low and high resistance states of M1 as ON1 and OFF1, respectively, to distinguish them from the total resistance states (HRS, IRS, and LRS) and similarly designated ON2 and OFF2 for M2.

For the direct transition between states, the specification of the two memristors should satisfy the following conditions. One (i.e., M1) should have a higher OFF1 resistance and a lower set voltage than the other (i.e., M2). Then, in the M1‐M2 serial configuration, when the applied voltage (*V*_App_) is increased from the HRS (i.e., the OFF1‐OFF2 states for M1‐M2), most of the *V*_App_ is applied to the M1 as *R*_OFF1_ >> *R*_OFF2_, and thus, M1 can be set‐switched first. Once the M1 becomes ON1, the *V*_App_ will be redistributed by a voltage divider. If the redistributed voltage across M2 is still lower than the set voltage of M2, the overall device state will be stable at ON1‐OFF2, which corresponds to the IRS. Then, increasing the *V*_App_ will eventually set‐switch the M2, resulting in ON1‐ON2, the LRS.

To realize the devised serial memristor configuration in a single device, we employed a conventional bipolar‐type Ta/TaO*_x_*/Pt memristor system^[^ ^35^, ^36^ ^]^ and formed the TaO*_x_* layer as two layers with different oxygen contents and layer thickness. We tried various combinations of oxygen contents and thickness of the two layers to optimize the system and found the optimum device stack (see Note SI and Figure S1 in the Supporting Information.)

**Figure 1:**
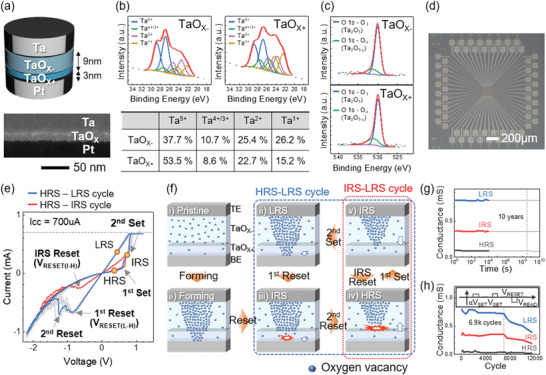
A ternary‐state bilayer tantalum oxide memristor device and its mechanism. a) The structure of the device and a cross‐section TEM. (Scale bar: 50 nm) b) Ta 4f peak fittings of the XPS spectrum of the TaO*_X_*_−_ and TaO*_X_*_+_ layers, and the atomic percentage of each component derived as a result. c) O 1s peak fittings of the XPS spectrum of the oxide layers. d) Optical microscopic image of the 16 × 16 array (Scale bar: 200 µm). e) The *I*–*V* characteristics of two switching cycles, one from HRS to LRS (blue) and the other from HRS to IRS (red). f) Schematic illustrations to show the oxygen vacancy distributions at each state. g) Retention characteristics of the LRS, IRS, and HRS at room temperature. h) Cycling endurance results. The inset shows the pulse timing sequence to read the ternary states.

**Figure** **1**a shows a schematic of the optimized Ta/TaO*_X_*_−_/TaO*x*_+_/Pt stack (top panel) and its transmission electron microscopy (TEM) image (bottom panel). The upper TaO*_X_*_−_ and lower TaO*_X_*_+_ layers were deposited with O_2_/Ar gas flow ratios of 0.15 and 0.3, and thicknesses of 9 and 3 nm, respectively. Due to the different O_2_ partial pressure during deposition, the two layers contained different amounts of oxygen in the film. The X‐ray photoelectron spectroscopy (XPS) analysis of the Ta 4f spectra determined that *X*− = ≈1.6 and *X*+ = ≈1.9 as shown in Figure 1b, meaning both layers were oxygen‐deficient and TaO*_X_*_−_ was more oxygen‐deficient than TaO*_X_*_+_. The energy levels of the four peaks related to Ta4f_7/2_ were 26.8 ± 0.1 eV for Ta^5+^, 25.3 ± 0.2 eV for Ta^4+/3+^, 23.6 ± 0.2 eV for Ta^2+^, and 22.3 ± 0.2 eV for Ta^1+^.^[^ ^37^, ^38^, ^39^, ^40^, ^41^, ^42^ ^]^ Each Ta 4f_5/2_ peak is 1.9 eV higher than each Ta 4f_7/2_ peak. The area ratio of the doublet was 1 (for 4f_5/2_) : 1.3 (for 4f_7/2_). The FWHM (full width at half maximum) of all peaks was applied equally with 1.73 eV. The bottom table panel shows the atomic percentage of each peak. The O 1s spectra of both layers are shown in Figure 1c, of which results are consistent with the Ta 4f spectra.^[^ ^43^, ^44^ ^]^ The blue peak located at 530 eV (O_I_) and the green peak at 531 eV (O_II_) are related to Ta—O bonding in a stoichiometric Ta_2_O_5_ and oxygen‐deficient one, respectively. The area ratio of O_I_ (blue)/O_II_ (green) is 3.57 in the TaO*_X_*_−_, and 5.0 in the TaO*_X_*_±_, confirming TaO*_X_*_−_ is more oxygen‐deficient.

We integrated the cell at the crossbar device to demonstrate the stateful logic. Figure 1d shows an optical microscopy image of the 16 × 16 crossbar array device. The line width at the cross‐point is 5 µm, so the device area is 5 x 5 µm^2^. Figure 1e shows the resistance switching *I*–*V* curves of the optimized ternary state memristor after electroforming with a 700 µA of compliance current (*I*_CC_). It shows a drastic set switching from HRS to IRS at 0.64 V, and from IRS to LRS at 0.78 V, allowing a clear distinction between states. For the convenience of calculation, we defined the higher set voltage leading to LRS as *V*_SET_ and the lower set voltage as *αV*_SET_ (0 < *α* < 1), where *α* = 0.82 in the device. The two set voltages distributions were not overlapped during cycling, suggesting a reliable stateful logic operation is possible (The variations of the set voltages are shown in Figure S2, Supporting Information). Also, it shows a two‐step reset process. One of the reset curves is highlighted, which shows a two‐step reset from LRS to at −0.8 V and from to HRS at −1.24 V. As the reset switching is gradual, the two‐step reset switching is less distinguishable than the two‐step set switching. In the stateful logic operation, only the first reset voltage is important, and the second one is unnecessary. Because once the reset switching is initiated, the device goes to the HRS spontaneously due to the node voltage increase by the voltage divider. For calculation, the reset voltage from LRS to HRS (*V*_RESET(L‐H)_) was normalized to −1.02 *V*_SET_. When the initial state was IRS, the reset voltage from IRS to HRS (*V*_RESET(I‐H)_) was decreased to −0.66 V as shown in Figure 1e, which was normalized to −0.84 *V*_SET_ for calculation. The reset voltage from the IRS is smaller than from the LRS because the filament in the IRS is weaker than the LRS. Also, the conductance of each state could be normalized to the conductance of the LRS; *G*_HRS_ is 0.1 *G*_LRS_, and *G*_IRS_ is 0.5 *G*_LRS_.

The double switching mechanism from the oxygen concentration‐modulated TaO*_X_*_−_/TaO*_X_*_+_ can be understood in Figure 1f.^[^ ^45^, ^46^, ^47^, ^48^, ^49^ ^]^ Figure 1f –i shows the pristine state of the device, where oxygen vacancies are drawn as blue dots. It shows a higher concentration of oxygen vacancies in the upper TaO*_X_*_−_ layer than in the lower TaO*_X_*_+_. After electroforming with a positive bias, a conical shape conducting filament was formed with a wider width at the upper side, as shown in Figure 1f ‐ii. With this filament configuration, the first reset switching destroys the filament in the bottom TaO*_X_*_+_ layer where the filament is the weakest (Figure 1f ‐iii). Then, additional reset switching ruptures the filament in the TaO*_X_*_−_ layer from the bottom, resulting in the complete HRS.(Figure 1f ‐iv) In the HRS, the 1st set switching at *αV*_SET_ can partially rejuvenate the filament only in the upper TaO*_X_*_−_ layer because it contains more oxygen vacancies so forms the filament easily. This leads to the IRS (ON1 of M1 and OFF2 of M2) as shown in Figure 1f ‐v. At the IRS, additional voltage to *V*_SET_ completely set‐switches M2, producing the LRS (Figure 1f ‐vi) again, which is the 2nd set switching. Or, the IRS can be reset switched to the HRS (Figure 1f ‐iv) by applying *V*_RESET(I‐H)_. The suggested model can also consistently explain other devices in Figure S1 (Supporting Information) made of various combinations of oxygen contents and thickness (see Note SI for more detailed discussion, Supporting Information). The conduction mechanism analysis results are included in Figure S3 (Supporting Information). All states showed ohmic or hopping conduction except for the Schottky conduction of HRS at high voltages, validating the proposed oxygen vacancy‐mediated switching model in Figure 1e.^[^ ^50^ ^]^

To confirm the stability of the device required for the ternary stateful logic operation, retention and endurance characteristics of the device were investigated. Figure 1g shows the retention of all states up to 10^4^ s in room temperature, suggesting the device is viable for the ternary stateful logic demonstration. Figure 1h shows the cycling endurance of the device. The inset shows a pulse cycle to read the ternary states. All states were constant up to 6900 cycles and degraded to the HRS gradually. Although both retention and endurance performance should be improved for practical use, they were sufficient to validate the ternary stateful logic for this study.

## 3 Ternary Stateful Logic Gate Investigation via Stateful Neural Network

**Figure 2:**
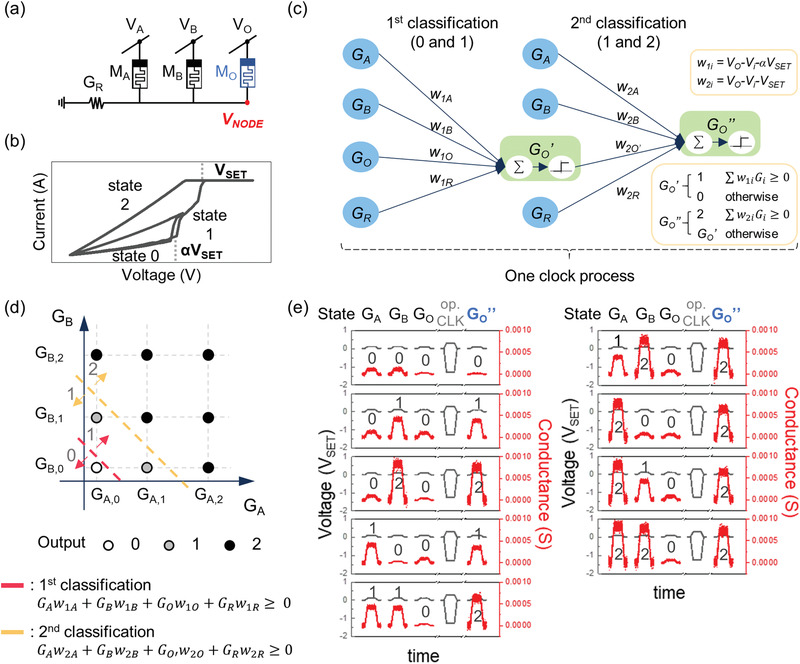
Ternary stateful logic and stateful neural network. a) Configuration of memristor cells for ternary stateful logic. Each memristor has three distinct states. b) Simplified *I*–*V* characteristics required for ternary stateful logic. It requires abrupt switching at the boundary of 0 and 1, and 1 and 2. c) A two‐layer ternary stateful neural network. In the first layer, the threshold is *αV*_SET_, and the 1st classification of 0 and 1 occurs. Even with the same voltage, in the second layer, the threshold changes (*V*_SET_) and the 2nd classification occurs, separating 1 and 2. d) Decision boundaries for strong conjunction of Łukasiewicz logic. e) Experimental demonstration of the strong disjunction gate.

We investigated viable ternary stateful logic gates using the developed ternary memristor. **Figure** **2**a shows a set of cells for executing the ternary stateful logic in the memristive crossbar array. The configuration and operating process are identical to conventional binary stateful logic, but have the ternary state cell characteristics. Figure 2b shows a representative *I*–*V* curve of the ternary state memristor. Here, the HRS, IRS, and LRS are defined as states 0, 1, and 2, respectively. A stable IRS can be obtained at the applied voltage between *αV*_SET_ and *V*_SET_. In this study, the output cell is initialized to HRS, and the logical output is obtained by a conditional (partial or full) set switching of it. Thus, the ternary gate operation would result in nonswitching (from 0 to 0) or partial switching (from 0 to 1) or full switching (from 1 to 2) of the output cell.

Sun et al. established a stateful neural network theory, expanded from the conventional binary stateful logic, a systematic method for finding possible stateful logic gates by drawing a decision boundary from the input state plot.^[^ ^9^, ^51^, ^52^ ^]^ We adopted this methodology to find the possible ternary stateful logic gates. The stateful neural network theory can be summarized as follows. For the three cell configuration in Figure 2a, the set switching condition for the output memristor (M_O_) is *V*_O_ − *V*_NODE_ ≥ *V*_th_, where $V_{\mathrm{NODE}}=\;\left(\sum_{i}G_{i}V_{i}/\sum_{i}G_{i}\right)$ according to Kirchhoff's current law, $\sum_{i}\left(V_{i}-V_{\mathrm{NODE}}\right)\;G_{i}=\;0$. By multiplying both sides by $\sum_{i}G_{i}$, which is always positive, the set switching condition can be organized to $\sum_{i}G_{i}w_{i}\geq 0$, where *w_i_* = *V*_O_  − *V*_i_ − *V*_th_. $\sum_{i}G_{i}w_{i}$ is the form of the weighted sum in the neural network, making the stateful logic a stateful neural network.

In the ternary state memristor, partial switching and full switching are sequential. Thus, the ternary gate operation, which utilizes both the partial and full switching, can be understood as two layers of the stateful neural network, as shown in Figure 2c. Here, *G*_A_ and *G*_B_ are two logical inputs in conductance, *G*_O_, *G*_O_’, and *G*_O_’’ are the initial, interim, and final conductance of the output cell and *G*_R_ is the conductance of the load resistor. Also, *w*_1*i*_ (where *i* = A, B, O, R) is the weight connecting G_i_ of the input neurons and *G*_O_’ of the output neuron in the first layer. Here, *w*_1*i*_ can be defined as *V*_O_ − *V_i_* − *V*_th,1_, where *V*_th,1_ is *αV*_SET_. Similarly, in the second layer, *w*_2*i*_ (where *i* = A, B, O’, R) is the weight connecting *G*_O_’ of the input neurons and *G*_O_’’ of the output neuron, and *V*_th,2_ is *V*_SET_.

Finding all of the operating voltage solutions for all ternary gates is a strenuous process. Before performing that, one can easily estimate if there are possible switching voltage solutions or not from the input state diagram, with decision boundary as shown in Figure 2d.^[^ ^6^ ^]^ In the diagram, two input conductances (*G*_A_ and *G*_B_) are assigned to the *x* and *y*‐axis, and the desired logical output values are plotted corresponding to the gate. *G*_A,0_, *G*_A,1_, and *G*_A,2_ (or *G*_B,0_, *G*_B,1_, and *G*_B,2_) are the three conductance states of the input cell M_A_ (or M_B_) corresponding to state 0, 1, and 2, respectively. The open, gray, and black symbols represent the final state 0, 1, and 2 of the output cells, respectively. The diagram in Figure 2d shows a strong disjunction gate (⊕) of Ł3 as an example, whose truth value can be expressed by ⊕(*G*_A_, *G*_B_) = MIN{2, *G*_A_ + *G*_B_}.^[^ ^17^ ^]^ By definition, the output of (*G*_A,0_, *G*_B,0_) should be 0, and the output of (*G*_A,1_, *G*_B,0_) and (*G*_A,0_, *G*_B,1_) should be 1. Otherwise, the output should be 2. Here, the switching condition equation ($\sum_{i}G_{i}w_{i}\;\geq$ 0) can be re‐arranged into a linear equation (*G*_B_ ≥ *aG*_A_ + *bG*_LRS_, where *a* and *b* are arbitrary values), which corresponds to the boundary line dividing the diagram into two sections with respect to the output states. Then, two decision boundaries can be drawn; the first one distinguishes the output state 0 or 1 (red line), and the other one does state 1 or 2 (yellow line).

As such, one can easily determine if any gate is viable or not just by drawing two boundary lines in the input state diagram as in Figure 2d. A detailed methodology is discussed in Note SII and Figures S3–S7 (Supporting Information).

After surveying all cases satisfying the conditions, we concluded that 551 ternary gates out of 19 683 were potentially possible in one operational clock. We called them potential ternary gates (PTG). The 551 PTG were obtained by just considering the final state of the output cell in the input state diagram and neglecting the change of inputs. Thus, the input boundary conditions should be additionally considered to confirm whether the gate operation would not destroy the inputs. That is, the applied voltages across the input memristor cells (*v*_M_) of 0 state should be lower than *αV*_SET_ (*v*_M_ < *αV*_SET_), an input of 1 should be higher than *V*_RESET(I‐H)_ and lower than *V*_SET_ (*V*_RESET(L‐H)_ < *v*_M_ < *V*_SET_), and an input of 2 should be higher than *V*_RESET(L‐H)_ (*V*_RESET(L‐H)_ < *v*_M_). After considering the input boundary conditions, we could determine that 157 gates satisfied both the input and output boundary conditions so were implementable in the given device. We called them “ternary unit gates (TUG)”. The number of TUG was sufficient to perform ternary computing (see Note SIII and Figures S8–S10 for more details, Supporting Information).

Figure 2e shows an experimental demonstration of one of the TUG, the strong disjunction gate. The black and red lines indicate the applied voltage pulses and the conductance of the cells, respectively. For better presentation, all voltages are normalized by *V*_SET_. After solving the inequality equations of the boundary conditions as shown in Figure S8 (Supporting Information), the operating pulse heights were selected to be −1.3 *V*_SET_ to *V*_A_ and *V*_B_, and 0.31 *V*_SET_ to *V*_O_ with 0.15 *G*_LRS_ to *G*_R_. The pulse widths at the maximum amplitude were 150 µs, and rise time, and fall time were equally 50 µs. At each panel, before and after applying the operating voltages, the initial states of *G*_A_, *G*_B_, and *G*_O_ and the final state of *G*_O_’’ were sequentially read by 0.1 *V*_SET_ of read pulse. This confirmed that the strong disjunction gate operation was successful.

## 4 Execution of Stateful Three‐Valued Łukasiewicz Logic and Full Adder

**Figure 3:**
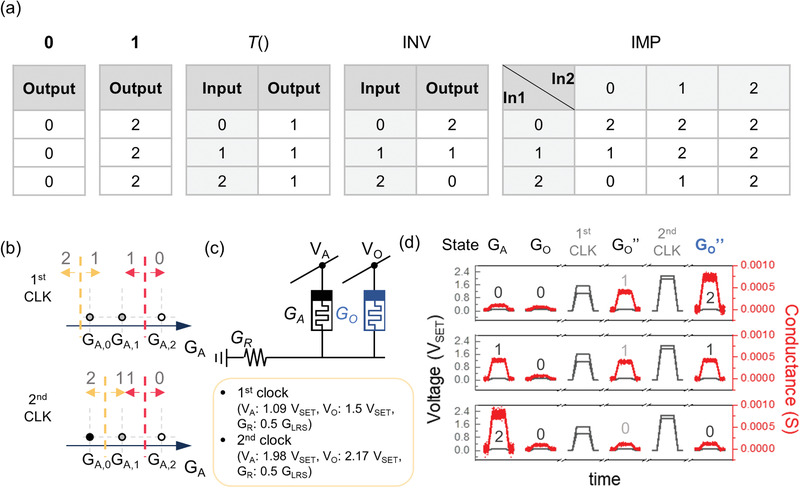
Functionally complete stateful three‐valued Łukasiewicz logic system and demonstration of inversion (INV) gate. a) Truth tables for the gates of a three‐valued functionally complete Łukasiewicz logic system. b) Input state diagram for the INV gate on each clock. c) One‐input‐one‐output configuration and required voltages for INV gate operation. d) Experimental operation of the INV gate. The read currents in read and verification steps, and operating voltages in execution steps are shown in red and black, respectively.

We experimentally demonstrated all of the required Łukasiewicz (Ł3) logic gates (i.e., **0**, **1**, INV, IMP) as well as the *T*() using the integrated device in Figure 1c.^[^ ^19^ ^]^ (Note that **0** (Bold zero) and **1** (Bold one) refer to the names of the gates that result in outputs 0 and 2, respectively.) The truth tables of the Ł3 gates and *T*() are shown in **Figure** **3**a. The **0**, **1**, and *T*() gates are initialization gates that program the output cell to state 0, 2, and 1, respectively, regardless of the inputs. Thus, they can be achieved by applying 0.1 *V*_SET_ for **0**, *V*_SET_ for **1**, and 0.82 *V*_SET_ for *T*(), as shown in Figure S11 (Supporting Information).

Figure 3b shows the input state diagram of the INV gate. The INV gate is a one‐input‐one‐output gate so that it needs only one dimension in the diagram. The INV gate belongs to the PTG but is not in the TUG, meaning the gate operation is possible for multiple clocks. Therefore, we executed it with two sequential clocks. Figure 3c,d shows the cell configuration and the experimental demonstration results, respectively. For the first clock, *V*_A_ and *V*_O_ were set to 1.09 *V*_SET_ and 1.5 *V*_SET_, respectively, which would result in a partial set switching of the output cell from 0 to 1, only if the input was 0 or 1. For the second clock, *V*_A_ and V_O_ were changed to 1.98 and 2.17 *V*_SET_, respectively, and it would switch the output cell from 1 to 2, only if the input was 0.

**Figure 4:**
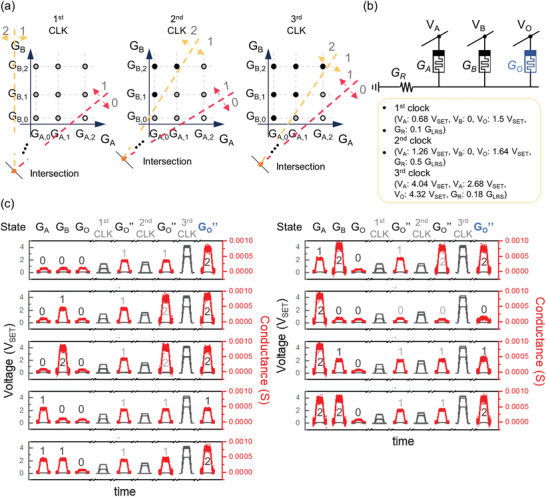
Demonstration of an Ł3 implication (IMP) gate. a) Input state diagrams of each of the three clocks for the IMP gate. Each step is included in the ternary unit gates. b) Two‐input‐one‐output configuration, and required voltages and resistors connected for each clock for the IMP gate operation. c) Experimental demonstration of the IMP gate for nine input cases of two ternary inputs. The read currents and operating voltages are shown in red and black, respectively.

Next, the IMP gate of Ł3 was investigated. The IMP gate is also included in PTG but not in TUG, and thus it is executable by three sequential clocks. **Figure** **4**a shows the input state diagram for the three clocks. Figure 4b shows the cell configuration for executing the IMP gate and the applied voltage conditions for each clock. Figure 4c shows the experimental data for all input conditions, proving the IMP gate operation was successful. In this way, 19 683 of all two‐input ternary gates can be realized through the cascading of Ł3 logic gates.

Next, we demonstrated a ternary full adder operation with the device. One of the advantages of stateful logic with a neural network is that it is easy to execute multi‐input (more than two) gates such as carry‐out and sum operations by selecting multiple word lines. In the binary stateful logic, both the carry‐out and sum operations were possible with one clock per each using three and four inputs.^[^ ^9^ ^]^ Similarly, we investigated the most compact carry‐out and sum operation sequences for executing the ternary full adder.

**Figure 5:**
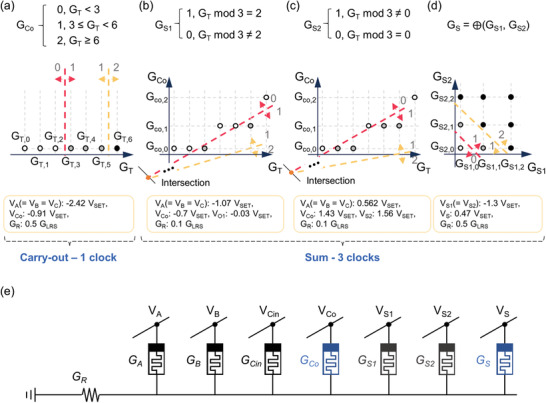
Stateful ternary full adder. Input state diagrams for a) carry‐out C_O_, b) S_1_ (first cache for sum), c) S_2_ (second cache for sum), and d) strong disjunction of Ł3 with G_S1_ and G_S2_ as inputs for the sum value, S. The carry‐out operation was done with one clock a) and the sum operation was done with three clocks b–d). e) Circuit configuration for full adder operation. Two extra caches (S_1_ and S_2_) are needed for the moment. The entire demonstration of the full adder can be found in Figure S13 (Supporting Information).

In our methodology, the first step in the gate investigation was to draw an input state diagram. However, multi‐input gates require multiple dimensional spaces to express all input cases, which is complicated. Therefore, we reduced the dimensions of the input states with the following treatment. We defined a new parameter *G*_T_ to be the sum of all inputs (*G*_T_ = *G*_A_ + *G*_B_ + *G*_Cin_), considering that the carry‐out value is associated with the sum of the inputs. In this way, the 27 input cases could be reduced to 7, assuming the conductance ratios were *G*_state0_ ≈ 0, *G*_state1_ = 0.5, and *G*_state2_ = 1.0 (see Figure S12 for the reduced truth table of the full adder, Supporting Information). Then, a 1D input state diagram can be drawn for the carry‐out operation, as in **Figure** **5**a. In this diagram, if *G*_T_ is greater than or equal to 3*G*_LRS_, the output is 1, and if *G*_T_ is greater than or equal to 6*G*_LRS_, the output is 2. The two boundary conditions satisfied the switching rules so that they were executable with one voltage clock.

Next, the sum operation required at least three clocks and seven cells (i.e., four inputs, two caches, and one output). The cache cells were initialized to 0 before the operation for temporarily storing intermediary values for the first and second clocks. Those cache values can be deleted after the computation process finishes. For the first and second clocks, a 2D input state diagram can be drawn using *G*_Co_ and *G*_T_ as the two inputs, where *G*_Co_ is the output of the carry‐out gate. Then, the decision boundaries for each clock can be drawn as in Figure 5b,c. With the first clock, the first cache was programmed to 1 only if a remainder of *G*_T_/3 was 2. With the second clock, the second cache was programmed to 1 if a remainder of *G*_T_/3 was 1 or 2. Then, with the third clock, the strong disjunction gate operation with two buffers as inputs can complete the sum operation. The entire demonstration of the full adder can be found in Figure S13 (Supporting Information). In summary, the ternary full adder required 7 cells (i.e., three inputs, one output for carry‐out, two caches for sum, and one output for sum) and 4 clocks (i.e., one for carry‐out and three for sum). Alternatively, if the caches are reset initialized for subsequent use, the total number of required cells and clocks can be 5 and 5, respectively.

The efficiency of the developed ternary full adder was compared to that of a binary full adder. For the measure of efficiency, we adopted a spatiotemporal cost (STC) which is the value multiplying the number of required core cells and the number of clocks.^[^ ^4^, ^53^ ^]^ For a fair comparison, it was considered an *N*‐digit decimal number calculation task. Also, it was assumed that all bits were located along the same row. In binary stateful logic, Sun et al. proposed that the binary stateful full adder can be executable with 5 cells and 2 clocks, which was the most efficient full adder sequence.^[^ ^9^ ^]^ In this case, the STC value of the two‐bit binary full adder is 10.

In an *n*‐bit full adder, the carry‐in value from the second bit comes from the carry‐out of the previous bit. So only the first bit needs 5 cells and the second and next bits need additional 4 cells. Thus, the total number of cells is 4*n*+1, while the number of clocks is 2*n*. This results in 8*n* ^2^+2*n* of the STC value of the *n*‐bit full adder. Similarly, the proposed ternary stateful logic requires 4*n*+1 cells and 4*n*+1 clocks including the reset initialization clock at the end (see Figure S14 for resetting the cache cells to be reused through one extra clock, Supporting Information). Considering that the *N*‐digit decimal number can be converted into an ≈3.32*N*‐digit binary number and an ≈2.10*N*‐digit ternary number, the STC value for the *N*‐digit decimal number can be ≈88.18*N^2^* +6.64*N* for binary logic and ≈70.56*N^2^* +16.80*N* for ternary logic. When *N* is high enough, the linear term will be negligible and the ternary logic will be more efficient, by about 20%. **Table** **1** summarizes the comparison.

**Table 1:** Comparison of the full adder execution in binary and ternary stateful neural networks

	Binary full adder (Sun et al.^[^ ^9^ ^]^)	Ternary full adder (This work)
Clocks / Cells / STC for *n* digit adder	2*n* / 4*n* + 1 / 2*n* (4*n* + 1)	4*n* + 1 / 4*n* + 1 / (4*n* + 1)^2^
Clocks / Cells / STC for addition of *N* **‐**digits decimal number	6.64*N* / 13.28*N* + 1 / ≈88.18*N^2^* + 6.64*N*	8.40*N* + 1 / 8.40*N* + 1 / ≈70.56*N^2^* + 16.80*N*

## 5 Conclusion

We fabricated a bilayered tantalum oxide memristor device that possessed discrete ternary states, and the switching between states was abrupt. This allowed us to implement ternary logic on the device via a ternary stateful neural network. Before the experimental demonstration, we investigated all possible ternary gates theoretically, utilizing an input state diagram and neural network‐based classification methodology. Consequently, we concluded that 157 gates were possible in this device. Their optimized combinations and multi‐input operation strategy ensured a functionally complete three‐valued Łukasiewicz logic and enabled a ternary full adder operation about 20% efficiently than the binary stateful logic. The computational efficiency can be further improved by device parameter optimization. For example, if the amplitudes of the two reset voltages and *αV*_SET_ are high enough, the sum operation can be possible with two clocks using *G*_T_, *G*_Co_, and *G*_S1_ as inputs.

Although this study showed the feasibility of the ternary stateful logic, there are still challenges to resolve before it can be practically used. One of the most crucial issues is switching reliability, which is also associated with device‐to‐device and cell‐to‐cell variations. To solve those issues, it may be better to develop and apply error‐correction methodologies for the ternary logic, similar to those employed with binary stateful logic.^[^ ^53^ ^]^

## 6 Experimental Section

### Device Fabrication

The Ta/TaO*_X_*_−_/TaO*_X_*_+_/Pt structure was fabricated using the following process. The bottom electrode (50 nm thick Pt) was deposited on a Ti(adhesion layer) /Si substrate by e‐beam evaporation after photoresist pattern formation, and it was patterned by lift‐off process. Afterward, two layers of tantalum oxide were deposited by reactive sputtering with various combinations of oxygen flow rates and thicknesses. The deposition conditions of the device used in this study were an Ar/O_2_ flow ratio of 0.15 and a thickness of about 9 nm for the TaO*_X_*_−_ layer, and an Ar/O_2_ flow ratio of 0.3 and a thickness of about 3 nm for the TaO*_X_*_+_ layer. Then, the top electrode (120 nm thick Ta and 50 nm thick Pt for passivation) was formed by e‐beam evaporation followed by lift‐off patterning.

### Electrical Measurements

All electrical testing was performed using a semiconductor analyzer (Keithley 4200A‐SCS) and a probe station. The *I*–*V* characteristics were obtained in a DC sweep using two SMUs (Source Measurement Unit). The bottom electrode was ground while the top electrode was biased, and the current was read at the top electrode with a sweep rate of 0.8 V s^−1^. For the gate operation demonstration, voltage pulses were applied using a Keithley 4225‐PMU (Pulse Measurement Unit) and 4225‐RPM (Remote Amplifier/Switch). The pulse width was 150 µs, and the rise time, fall time, delay time, and hold time were all 50 µs.

## Conflict of Interest

The authors declare no conflict of interest.

## Supporting information

Supporting Information

## Data Availability

The data that support the findings of this study are available from the corresponding author upon reasonable request.

## References

[1] D. Ielmini , H. S. P. Wong , Nat. Electron. 2018, 1, 333.

[2] M. A. Zidan , J. P. Strachan , W. D. Lu , Nat. Electron. 2018, 1, 22.

[3] D. S. Jeong , K. M. Kim , S. Kim , B. J. Choi , C. S. Hwang , Adv. Electron. Mater. 2016, 2, 1600090.

[4] Y. S. Kim , M. W. Son , K. M. Kim , Adv. Intell. Syst. 2021, 3, 2000278.

[5] A. Siemon , T. Breuer , N. Aslam , S. Ferch , W. Kim , J. Van Den Hurk , V. Rana , S. Hoffmann‐Eifert , R. Waser , S. Menzel , E. Linn , Adv. Funct. Mater. 2015, 25, 6414.

[6] L. Liu , Y. Li , X. Huang , J. Chen , Z. Yang , K. H. Xue , M. Xu , H. Chen , P. Zhou , X. Miao , Adv. Sci. 2021, 8, 2005038. 10.1002/advs.202005038 PMC8336485 34050639

[7] T. You , Y. Shuai , W. Luo , N. Du , D. Bürger , I. Skorupa , R. Hübner , S. Henker , C. Mayr , R. Schüffny , T. Mikolajick , O. G. Schmidt , H. Schmidt , Adv. Funct. Mater. 2014, 24, 3357.

[8] J. Borghetti , G. S. Snider , P. J. Kuekes , J. J. Yang , D. R. Stewart , R. S. Williams , Nature 2010, 464, 873. 20376145 10.1038/nature08940

[9] Z. Sun , E. Ambrosi , A. Bricalli , D. Ielmini , Adv. Mater. 2018, 30, 1802554. 10.1002/adma.201802554 30079525

[10] K. M. Kim , R. S. Williams , IEEE Trans. Circuits Syst. I Regul. Pap. 2019, 66, 4348.

[11] K. M. Kim , N. Xu , X. Shao , K. J. Yoon , H. J. Kim , R. S. Williams , C. S. Hwang , Phys. Status Solidi RRL 2019, 13, 1800629.

[12] Y. S. Kim , M. W. Son , H. Song , J. Park , J. An , J. B. Jeon , G. Y. Kim , S. Son , K. M. Kim , Adv. Intell. Syst. 2020, 2, 1900156.

[13] L. Cheng , Y. Li , K. Yin , S. Hu , Y. Su , M. Jin , Z. Wang , T. Chang , X. Miao , Adv. Funct. Mater. 2019, 29, 1905660.

[14] P. Huang , J. Kang , Y. Zhao , S. Chen , R. Han , Z. Zhou , Z. Chen , W. Ma , M. Li , L. Liu , X. Liu , Adv. Mater. 2016, 28, 9758. 27717010 10.1002/adma.201602418

[15] N. Xu , T. G. Park , H. J. Kim , X. Shao , K. J. Yoon , T. H. Park , L. Fang , K. M. Kim , C. S. Hwang , Adv. Intell. Syst. 2020, 2, 1900082.

[16] K. J. Yoon , J. W. Han , W. Bae , Adv. Electron. Mater. 2020, 6, 2000672.

[17] S. Gottwald , “Many‐Valued Logic,” https://plato.stanford.edu/entries/logic-manyvalued/ (accessed: November 2021).

[18] J. Łukasiewicz , Ruch Filoz. 1920, 5, 169.

[19] D. M. Miller , M. A. Thornton , Multiple Valued Logic: Concepts and Representations, Morgan & Claypool, San Rafael, CA 2007.

[20] J. Slupecki , Comptes rendus des séances la Société des Sciences et des lettres Varsovie, 1936, 29, 9.

[21] J. Slupecki , L. Borkowski , Elements of Mathematical Logic and Set Theory, Pergamon Press, Oxford, New York 1967.

[22] J. W. Jeong , Y. E. Choi , W. S. Kim , J. H. Park , S. Kim , S. Shin , K. Lee , J. Chang , S. J. Kim , K. R. Kim , Nat. Electron. 2019, 2, 307.

[23] J. Shim , S. Oh , D. H. Kang , S. H. Jo , M. H. Ali , W. Y. Choi , K. Heo , J. Jeon , S. Lee , M. Kim , Y. J. Song , J. H. Park , Nat. Commun. 2016, 7, 13413. 27819264 10.1038/ncomms13413 PMC5103069

[24] G. Liu , S. Ahsan , A. G. Khitun , R. K. Lake , A. A. Balandin , J. Appl. Phys. 2013, 114, 154310.

[25] A. Nourbakhsh , A. Zubair , M. S. Dresselhaus , T. Palacios , Nano Lett. 2016, 16, 1359. 26784325 10.1021/acs.nanolett.5b04791

[26] L. Lee , J. Hwang , J. W. Jung , J. Kim , H. I. Lee , S. Heo , M. Yoon , S. Choi , N. Van Long , J. Park , J. W. Jeong , J. Kim , K. R. Kim , D. H. Kim , S. Im , B. H. Lee , K. Cho , M. M. Sung , Nat. Commun. 2019, 10, 1998. 31040277 10.1038/s41467-019-09998-x PMC6491477

[27] X. Y. Wang , P. F. Zhou , J. K. Eshraghian , C. Y. Lin , H. H. C. Iu , T. C. Chang , S. M. Kang , IEEE Trans. Circuits Syst. I Regul. Pap. 2021, 68, 264.

[28] T. Breuer , L. Nielen , B. Roesgen , R. Waser , V. Rana , E. Linn , Sci. Rep. 2016, 6, 23967. 27046279 10.1038/srep23967 PMC4820708

[29] Y. J. Zhang , X. H. Chen , Z. R. Wang , Q. L. Chen , G. Liu , Y. Li , P. J. Wang , R. W. Li , X. S. Miao , IEEE Trans. Electron Devices 2019, 66, 4710.

[30] W. Xue , Y. Li , G. Liu , Z. Wang , W. Xiao , K. Jiang , Z. Zhong , S. Gao , J. Ding , X. Miao , X. H. Xu , R. W. Li , Adv. Electron. Mater. 2020, 6, 1901055.

[31] L. Cheng , H. X. Zheng , Y. Li , T. C. Chang , S. M. Sze , X. Miao , IEEE Trans. Electron Devices 2020, 67, 1293.

[32] W. Kim , A. Chattopadhyay , A. Siemon , E. Linn , R. Waser , V. Rana , Sci. Rep. 2016, 6, 36652. 27834352 10.1038/srep36652 PMC5105152

[33] D. Bhattacharjee , W. Kim , A. Chattopadhyay , R. Waser , V. Rana , Sci. Rep. 2018, 8, 2. 29311689 10.1038/s41598-017-18329-3 PMC5758575

[34] J. Reuben , R. Ben‐Hur , N. Wald , N. Talati , A. H. Ali , P.‐E. Gaillardon , S. Kvatinsky , in 2017 27th Int. Symp. Power Timing Model Optim. Simul., IEEE, Thessaloniki 2017, pp. 1–8.

[35] J. J. Yang , M.‐X. Zhang , J. P. Strachan , F. Miao , M. D. Pickett , R. D. Kelley , G. Medeiros‐Ribeiro , R. S. Williams , Appl. Phys. Lett. 2010, 97, 232102.

[36] M. J. Lee , C. B. Lee , D. Lee , S. R. Lee , M. Chang , J. H. Hur , Y. B. Kim , C. J. Kim , D. H. Seo , S. Seo , U. I. Chung , I. K. Yoo , K. Kim , Nat. Mater. 2011, 10, 625. 21743450 10.1038/nmat3070

[37] R. Simpson , R. G. White , J. F. Watts , M. A. Baker , Appl. Surf. Sci. 2017, 405, 79.

[38] G. S. Park , Y. B. Kim , S. Y. Park , X. S. Li , S. Heo , M. J. Lee , M. Chang , J. H. Kwon , M. Kim , U. I. Chung , R. Dittmann , R. Waser , K. Kim , Nat. Commun. 2013, 4, 2382. 24008898 10.1038/ncomms3382

[39] Y. R. Denny , T. Firmansyah , S. K. Oh , H. J. Kang , D. S. Yang , S. Heo , J. G. Chung , J. C. Lee , Mater. Res. Bull. 2016, 82, 1.

[40] M. V. Ivanov , T. V. Perevalov , V. S. Aliev , V. A. Gritsenko , V. V. Kaichev , J. Appl. Phys. 2011, 110, 024115.

[41] O. Kerrec , D. Devilliers , H. Groult , P. Marcus , Mater. Sci. Eng. B 1998, 55, 134.

[42] J. F. Moulder , W. F. Stickle , P. E. Sobol , K. D. Bornben , Handbook of X‐Ray Photoelectron Spectroscopy, Perkin‐Elmer Corporation Physical Electronics Division, Eden Prairie 1992.

[43] Z. Zhang , D. Luo , G. Li , R. Gao , M. Li , S. Li , L. Zhao , H. Dou , G. Wen , S. Sy , Y. Hu , J. Li , A. Yu , Z. Chen , Matter 2020, 3, 920.

[44] T. Kim , G. Baek , S. Yang , J. Y. Yang , K. S. Yoon , S. G. Kim , J. Y. Lee , H. S. Im , J. P. Hong , Sci. Rep. 2018, 8, 8532. 29867108 10.1038/s41598-018-26997-y PMC5986858

[45] K. M. Kim , S. R. Lee , S. Kim , M. Chang , C. S. Hwang , Adv. Funct. Mater. 2015, 25, 1527.

[46] K. M. Kim , C. S. Hwang , Appl. Phys. Lett. 2009, 94, 122109.

[47] J. P. Strachan , A. C. Torrezan , F. Miao , M. D. Pickett , J. Joshua Yang , W. Yi , G. Medeiros‐Ribeiro , R. Stanley Williams , IEEE Trans. Electron Devices 2013, 60, 2194.

[48] K. M. Kim , B. J. Choi , Y. C. Shin , S. Choi , C. S. Hwang , Appl. Phys. Lett. 2007, 91, 7.

[49] K. M. Kim , T. H. Park , C. S. Hwang , Sci. Rep. 2015, 5, 7844. 25598439 10.1038/srep07844 PMC4297972

[50] E. W. Lim , R. Ismail , Electron 2015, 4, 586.

[51] W. S. McCulloch , W. Pitts , Bull. Math. Biophys. 1943, 5, 115.

[52] R. Rojas , Neural Networks: A Systemic Introduction, Springer‐Verlag, Berlin 1996.

[53] J. H. In , Y. S. Kim , H. Song , G. M. Kim , J. An , J. B. Jeon , K. M. Kim , Adv. Intell. Syst. 2020, 2, 2000081.

