# Supplementary material for: Ternary Logic with Stateful Neural Networks Using a Bilayered TaO X ‐Based Memristor Exhibiting Ternary States
Source: Adv Sci (Weinh). 2021 Dec 16;9(5):2104107. doi: 10.1002/advs.202104107 (PMC8844464; doi:10.1002/advs.202104107)
Supplement: Supplementary file 1 — Supporting Information [file ADVS-9-2104107-s001.pdf]

WILEY-VCH

ADVANCED SCIENCE
Open Access

Supporting Information

for *Adv. Sci.,* DOI: 10.1002/advs.202104107

Ternary logic with Stateful Neural Networks using a bilayered TaO_X-based memristor exhibiting ternary states

*Young Seok Kim, Jangho An, Jae Bum Jeon, Myeong Won Son, Seoil Son, Woojoon Park, Younghyun Lee, Juseong Park, Geun Young Kim, Gwangmin Kim, Hanchan Song and Kyung Min Kim**

Supporting Information

Ternary logic with Stateful Neural Networks using a bilayered TaO_X-based memristor exhibiting ternary states

*Young Seok Kim, Jangho An, Jae Bum Jeon, Myeong Won Son, Seoil Son, Woojoon Park, Younghyun Lee, Juseong Park, Geun Young Kim, Gwangmin Kim, Hanchan Song and Kyung Min Kim**

Supporting Note Ⅰ.

Experimental results of devices fabricated under various conditions

We explored various combinations of bilayers to find the ternary-state memristor. Figure S1 shows the switching curves fabricated under various deposition conditions. In each panel, oxygen/argon gas flow ratio and film thickness for the TaO_{X-} and TaO_{X+} layers are denoted.

First, the oxygen partial pressure (P_O) for the double layers was investigated to show the ternary states as shown in Figure S1a. In our previous results, P_O = 0.3 was the optimum condition for a single layer of TaOx memristor. For the double layer device, we reduced the P_O of the upper layer from 0.175 to 0.125. Interestingly, at 0.175 and 0.15 of P_O, they showed a discrete intermediate state that may allow the ternary states of the memristor. At the lower P_O, it showed again a binary memristive behavior because the upper layer became metallic so that it worked as a series resistor.

After the oxygen partial pressures were fixed, we examined the optimized thickness combinations for the two layers suitable for the ternary logic operation. The required property for the ternary memristor is that the conductance of the IRS is the half of the conductance of the LRS and the conductance of the HRS is close to 0. The target condition could be achieved by the thickness optimization. Figure S1b shows the thickness dependence of the TaO_{X+} layer. As the thickness of the TaO_{X+} decreased, both the conductance of the IRS and HRS were increased. It inferred that the lower layer modulated the HRS and IRS simultaneously. To reduce the conductivity of the HRS while keeping the IRS, the upper layer thickness was increased.(Figure S1c) Eventually, at the upper layer thickness of 9 nm, the conductivity of the HRS was suppressed, and the desired conditions were obtained.

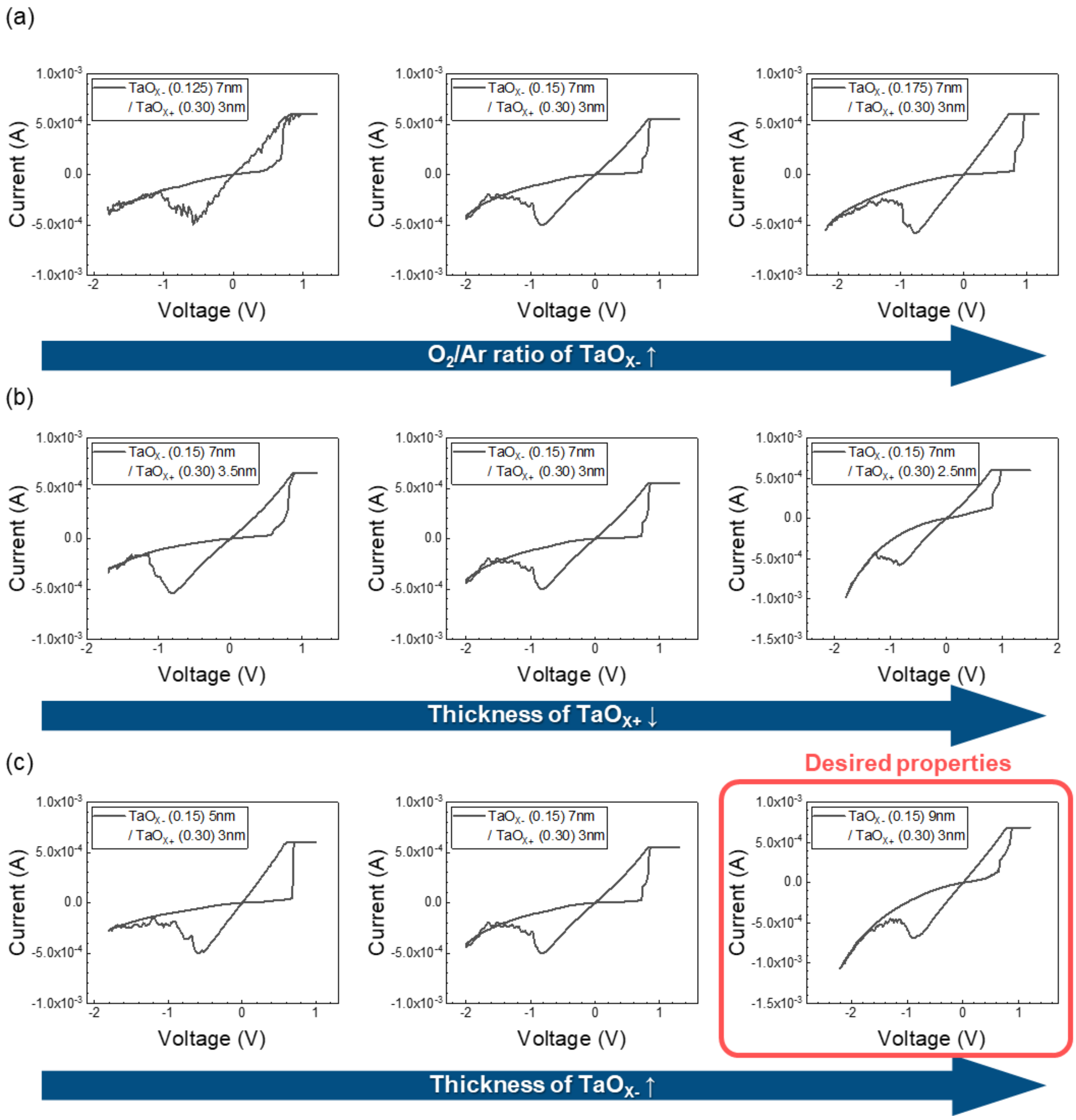

Figure S1 IV characteristics of devices manufactured while changing specific conditions. Based on the reference device, TaO_{X-} (0.15) 7nm / TaO_{X+} (0.30) 3nm, the devices were fabricated by modifying a) O_2/Ar flow ratio during sputtering TaO_{X-}, b) thickness of TaO_{X+}, and c) thickness of TaO_{X-}.

Based on the previous results, the overall switching mechanism could be understood by a serial connection of two distinct memristors. The voltage divider effect between two memristors determines the applied voltages at each node. Since TaO_{X-} has more oxygen vacancies and is thicker than TaO_{X+}, TaO_{X-} switches at a lower positive voltage. At αV_{SET}, TaO_{X-} is partially set-switched, and the device reaches the IRS.[1] Then the IRS resistance is close to the OFF2 resistance of the TaO_{X+}. Therefore, the OFF2 resistance is crucial, which is

determined by the thickness of the TaO_{X+}.[2] At V_{SET}, TaO_{X+} is set-switched, making both the TaO_{X-} and TaO_{X+} switched and thus the LRS.

At a negative voltage, TaO_{X+} resets prior to the TaO_{X-}, because the filament close to the anode is the weakest.[3] Afterward, reset occurs in the TaO_{X-} layer. Due to the gradual reset characteristics, the two-stage reset process is less clearly observed within the IV curve. Figure S2 shows the simulated set and reset switching curves of IRS and LRS using the tantalum oxide memristor model.[4]

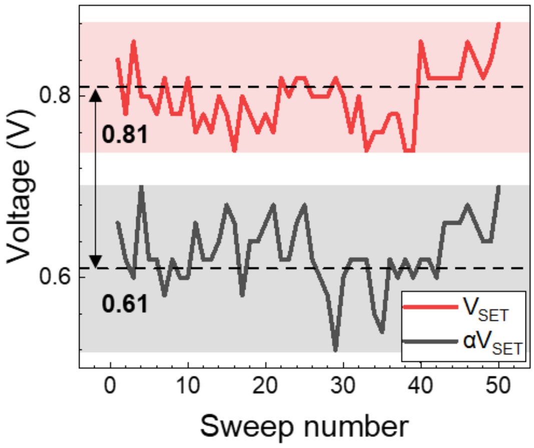

Figure S2 The variation of the first and second set voltages. The set voltages were 0.61 ± 0.09 V and 0.81 ± 0.07 V for the first and second set voltage, respectively, during 50 cycles. Although there were some switching voltage variations, they were not overlapped and the stateful logic operation is possible with a precise pulse condition control.

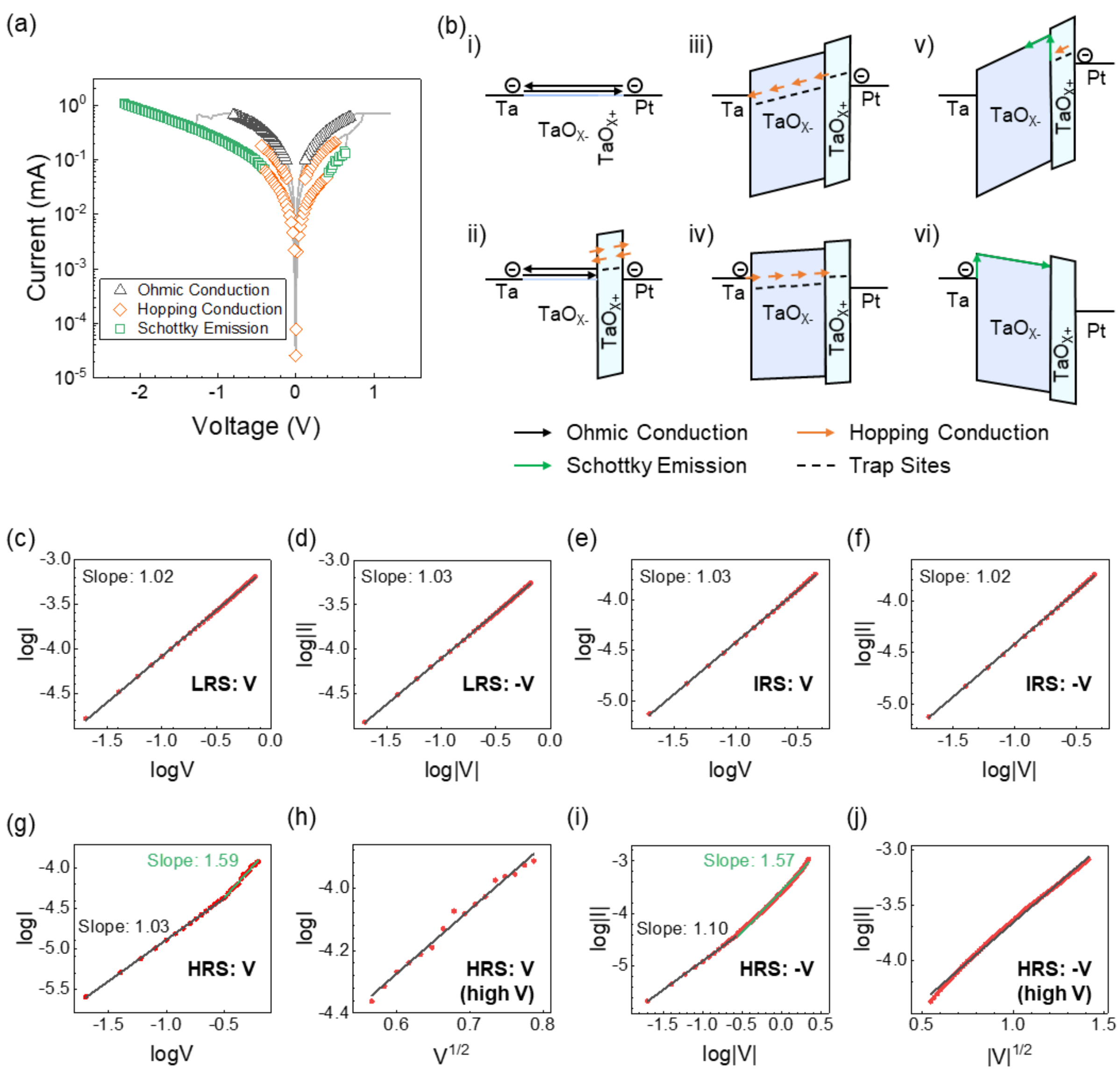

Figure S3 Conduction mechanism of the ternary-state bilayer tantalum oxide memristor device. a) The I-V curves of the device show the conduction mechanism for each state. b) Band diagrams and the dominant conduction path of each state: i) LRS, ii) IRS, iii) HRS at low positive voltages, iv) HRS at low negative voltages, v) HRS at high positive voltages, and vi) HRS at high negative voltages. c,d) The log I- log V plot of the LRS at the c) positive and d) negative voltages. e,f) Those of IRS at the e) positive and f) negative voltages. Considering the IRS is an insulating state, it can be reasonably understood as hopping conduction. g,h) The log I- log V plot of the HRS at the g) positive and h) negative voltages. They fits well only at low voltages. i,j) The log I - $V^{1/2}$ plot for Schottky conduction at high voltages of i) positive and j) negative voltages.

Supporting Note Ⅱ.

A detailed methodology for investigating the potential ternary gates (PTG) with a single clock considering only the output boundary lines

For the gates to be valid in the input state diagram, the following two rules should be satisfied to evaluate the possibility of the gates geometrically.

First, a set of full switching conditions should be a subset of a set of partial switching conditions at the input state diagram because the partial switching (from 0 to 1) is always preceded the full switching (from 1 to 2). Let define sets of inputs resulting in the same output as follows. At the first layer, two sets are possible; the input sets resulting in the output 0 can be expressed as $X_{1,0} = \{(G_A, G_B) \mid G_{O'} == 0\}$ and the input sets resulting in the output 1 can be $X_{1,1} = \{(G_A, G_B) \mid G_{O'} == 1\}$. At the second layer, those resulting in 1 and 2 can be $X_{2,1} = \{(G_A, G_B) \mid G_{O''} == 1\}$ and $X_{2,2} = \{(G_A, G_B) \mid G_{O''} == 2\}$, respectively. Then, $X_{1,1}$ should include $X_{2,2}$ ($X_{1,1} \supset X_{2,2}$). (See Figure S5 for an acceptable and unacceptable example because of the switching sequence).

Second, the voltage solutions enabling the gate operation should be present, and it can be geometrically determined and examined in the input state diagram. For the ease of convenience, we examine all ternary logic gates via three categories according to the number of inputs: 0, 1, and 2. All output cells before the operation were assumed to be in HRS.

i) Zero-input gates

They are three initialization gates making the output to 0, 1, or 2 states. Figure S6a shows the input state diagram of the gates that the output is independent of the input states; $G_{A,i}$ (or $G_{B,i}$) is the conductance of the input cell M_A (or M_B) when state is *i*.

ii) One-input gates

For these gate, only one input (G_A or G_B) is needed, and the other one is unnecessary. Thus, the slope of the boundary line is infinite or zero. For example, when G_A is the input, the slope is infinite, and there can be 4 possible options for the 1st decision boundary line in the

input state diagram, as shown in Figure S6b and S6c: $G_A < G_{HRS}$, $G_{HRS} < G_A < G_{IRS}$, $G_{IRS} < G_A < G_{LRS}$ and $G_A > G_{LRS}$. According to the first rule, the 2nd boundary is limitedly selectable by the 1st boundary. Finally, we need to confirm whether these choices can be consistent with ternary stateful neural networks, and for this, we consider the following four inequalities:

For the first boundary line;

$$G_A \geq aG_{LRS} \quad \text{(S1a)}$$

$$w_{1A}G_A + w_{1O}G_{HRS} + w_{1R}G_R \geq 0 \quad \text{(S1b)}$$

For the second boundary line;

$$G_A \geq bG_{LRS} \quad \text{(S2a)}$$

$$w_{2A}G_A + w_{2O}G_{IRS} + w_{2R}G_R \geq 0 \quad \text{(S2b)}$$

, where the coefficient a and b are arbitrary numbers. Inequality (S1a) and (S2a) refer to the decision boundaries at the input state diagram, and Inequality (S1b) and (S2b) refer to the weighted sum inequalities of the ternary stateful neural network. Thus, Inequality (S1a) and (S1b), and (S2a) and (S2b) should be matched, respectively. Substituting the definition $w_{ji} = V_O - V_i - V_{th,j}$, where $V_{th,1}$ is αV_{SET} and $V_{th,2}$ is V_{SET}, the following two equations are obtained:

$$w_{1O}G_{HRS} + w_{1R}G_R = -a \times w_{1A}G_{LRS} \quad \text{(S3a)}$$

$$w_{2O}G_{IRS} + w_{2R}G_R = -b \times w_{2A}G_{LRS} \quad \text{(S3b)}$$

Since there are two variables (V_A and V_O) and two equations, one can find their solution.

$$V_A = \frac{(-\alpha(G_{HRS}+a\times G_{LRS}+G_R)(b\times G_{LRS}+G_R)+(a\times G_{LRS}+G_R)(G_{IRS}+b\times G_{LRS}+G_R))V_{SET}}{(a-b)G_{LRS}\times G_R} \quad \text{(S4a)}$$

$$V_O = \frac{(-\alpha\times b(G_{HRS}+a\times G_{LRS}+G_R)+a(G_{IRS}+b\times G_{LRS}+G_R))V_{SET}}{(a-b)G_R} \quad \text{(S4b)}$$

The number of possible combinations of selecting two boundary lines among the 4 options with repetition allowed is ${}_4H_2$ (= ${}_5C_2$), 10, using the fomular of combination with repetition. Among them, three are corresponding to the zero-input gates so that they should be excluded. Conseqently, there are 7 sectioning cases. Once two boundary lines are chosen, the three domains divided by the two boundary lines can be assigned to the output values in ascending order (e.g., 0, 1, 2) or descending order (e.g., 2, 1, 0), considering the first rule of investigating PTG in page 6. In conclusion, 14 gates (=2×(${}_4H_2$ - 3)) are possible as G_A is the input. Similarly, when G_B is the input, another 14 gates are possible, making 28 one-input gates in total.

iii) Two-input gates

Both G_A and G_B are inputs so that the slope of the boundary lines can be from 0 to infinity. Then, similar to the one-input gate, one can get the following system of inequalities and the solution can be obtained by solving them.

For the first boundary line;

$$aG_A + G_B \geq bG_{LRS} \quad \text{(S5a)}$$

$$w_{1A}G_A + w_{1B}G_B + w_{1O}G_{HRS} + w_{1R}G_R \geq 0 \quad \text{(S5b)}$$

For the second boundary line;

$$cG_A + G_B \geq dG_{LRS} \quad \text{(S6a)}$$

$$w_{2A}G_A + w_{2B}G_B + w_{2O}G_{IRS} + w_{2R}G_R \geq 0 \quad \text{(S6b)}$$

, where coefficient a, b, c, and d are arbitrary numbers. For simplification, the coefficient of

G_B in Inequality (S5a) and (S6a) is set to 1.

Comparing the coefficents of (S5a) and (S5b) for making them equal, the following equations can be obtained:

$$w_{1A} = aw_{1B} \tag{S7a}$$

$$w_{1O}G_{HRS} + w_{1R}G_R = -b \times G_{LRS}w_{1B} \tag{S7b}$$

Similarly, for (S6a) and (S6b):

$$w_{2A} = cw_{2B} \tag{S7c}$$

$$w_{2O}G_{IRS} + w_{2R}G_R = -d \times G_{LRS}w_{2B} \tag{S7d}$$

There are three variables (V_A, V_B and V_O) that needed to be obtained in the four equations, and thus it is not possible to solve it as is. For this reason, we assumed two cases for further simplification. First, it is assumed that $V_A = V_B$, which is the case where the output does not care about the order of inputs. Then, the coefficients *a* and *c* are 1, making Equations (S7a) and (S7c) the same.

After the simplification, the solution can be written by the following equation.

$$V_A = V_B = \frac{(-\alpha(G_{HRS}+b\times G_{LRS}+G_R)(d\times G_{LRS}+G_R)+(b\times G_{LRS}+G_R)(G_{IRS}+d\times G_{LRS}+G_R))V_{SET}}{(b-d)G_R\times G_{LRS}} \tag{S8a}$$

$$V_O = \frac{(-\alpha\times d(G_{HRS}+b\times G_{LRS}+G_R)+b(G_{IRS}+d\times G_{LRS}+G_R))V_{SET}}{(b-d)G_R} \tag{S8b}$$

The results suggest that the intercepts (coefficient *b* and *d*) of the two boundary lines are arbitrary while its slope (coefficient *a* and *c*) should be -1. The 1st decision boundary has 6

possible options for intercept ($G_A + G_B$) in the diagram, as shown in Figure S6d: $G_A + G_B < \sqrt{2}G_{HRS}$, $\sqrt{2}G_{HRS} < G_A + G_B < (G_{HRS} + G_{IRS})/\sqrt{2}$, $(G_{HRS} + G_{IRS})/\sqrt{2} < G_A + G_B < \sqrt{2}G_{IRS}$, $\sqrt{2}G_{IRS} < G_A + G_B < (G_{IRS} + G_{LRS})/\sqrt{2}$, $(G_{IRS} + G_{LRS})/\sqrt{2} < G_A + G_B < \sqrt{2}G_{LRS}$ and $G_A + G_B > \sqrt{2}G_{LRS}$. The number of possible two-input gates corresponding to this case can be obtained similarly to one-input gates excluding the three zero-input gates. Since there are 6 options, the result is $2\times({}_6H_2 - 3)$, 36.

Second, it is assumed that $V_A \neq V_B$. In this case, the number of inequality equations can be reduced through the equivalence of Equation (S7a) and (S7c), or Equation (S7b) and (S7d). In the former case, considering that Equation (S7a) and (S7c) should be equivalent, $w_{1A} - aw_{1B} = w_{2A} - cw_{2B}$. Then, coefficient c can be expressed to $\frac{a\times w_{1B}+(1-\alpha)V_{SET}}{w_{2B}}$, and the solution can be obtained by combining Equations S7a, S7b and S7d.

$$V_A = \frac{(a\times G_R\left(\begin{matrix} G_{IRS}+G_R+d\times G_{LRS} \\ -\alpha(G_{HRS}+G_R+d\times G_{LRS}) \end{matrix}\right)+G_{LRS}\left(\begin{matrix} b(G_{IRS}+G_R+d\times G_{LRS}) \\ -\alpha(d\times G_{HRS}+b\times G_R+b\times d\times G_{LRS}) \end{matrix}\right))V_{SET}}{(b-d)G_R\times G_{LRS}} \quad \text{(S9a)}$$

$$V_B = \frac{(-\alpha(G_{HRS}+G_R+bG_{LRS})(G_R+d\times G_{LRS})+(G_R+b\times G_{LRS})(G_{IRS}+G_R+d\times G_{LRS}))V_{SET}}{(b-d)G_R\times G_{LRS}} \quad \text{(S9b)}$$

$$V_O = \frac{(-\alpha\times d(G_{HRS}+G_R+b\times G_{LRS})+b(G_{IRS}+G_R+d\times G_{LRS}))V_{SET}}{(b-d)G_R} \quad \text{(S9c)}$$

The boundary lines in the input state diagram can be written as follows:

$$aG_A + G_B \geq bG_{LRS} \quad \text{(S10a)}$$

$$\frac{a\times w_{1B}+(1-\alpha)V_{SET}}{w_{2B}}G_A + G_B \geq dG_{LRS} \quad \text{(S10b)}$$

It gives the intersection of the two decision boundaries to:

Intersection point (G_A, G_B) =

$$\left(-\frac{(b\times G_{LRS}-d\times G_{LRS})(V_B-V_O+V_{SET})}{(-1+a)(-1+\alpha)V_{SET}},\ \frac{G_{LRS}\,(-(-1+\alpha)b\times V_{SET}-a\times d(V_B-V_O+V_{SET})+a\times b(V_B-V_O+\alpha\times V_{SET}))}{(-1+a)(-1+\alpha)V_{SET}}\right)$$

(S11)

Substituting Equations (S9a), (S9b), and (S9c) into Equation (S11), and then the intersection point condition can be obtained:

$$G_A + G_B = -G_R + \frac{G_{IRS} - \alpha \times G_{HRS}}{(\alpha - 1)}. \quad \text{(S12)}$$

Equaton (S12) means that the intersection point lies on $G_B = -G_A - G_R + \frac{G_{IRS} - \alpha \times G_{HRS}}{(\alpha - 1)}$ in the input state diagram, which is called an intersection trajectory line. In conclusion, the intersection point of two boundary lines should lie on the intersection trajectory line.

In the latter case, equivalence of Equation (S7b) and (S7d), $w_{1O} G_{HRS} + w_{1R} G_R + b \times G_{LRS} w_{1B} = w_{2O} G_{IRS} + w_{2R} G_R + d \times G_{LRS} w_{2B}$, and coefficient d can be expressed to $\frac{b \times G_{LRS} \times w_{1B} + (-G_R - G_{IRS} + \alpha (G_{HRS} + G_R)) V_{SET}}{G_{LRS} \times w_{2B}}$, and the solution can be obtained by combining Equations (S7a), (S7b) and (S7c).

$$V_A = \frac{(a(-1+c)G_R + a \times \alpha (G_{HRS} + G_R - c \times G_L) + \alpha \times b \times G_{LRS} + b(-1+c)G_{LRS} - \alpha \times c (G_{HRS} + b \times G_{LRS}))V_{SET}}{(a-c)G_R}$$

(S13a)

$$V_B = \frac{(a \times \alpha \times G_{HRS} + (-1+c)(G_R + b \times G_{LRS}) + \alpha \left(G_R + b \times G_{LRS} - c(G_{HRS} + G_R + b \times G_{LRS})\right))V_{SET}}{(a-c)G_R}$$

(S13b)

$$V_O = \frac{(a \times \alpha (G_{HRS} + G_R) + \alpha \times b \times G_{LRS} + b(-1+c)G_{LRS} - \alpha \times c (G_{HRS} + G_R + b \times G_{LRS}))V_{SET}}{(a-c)G_R}$$

(S13c)

The boundary lines in the input state diagram can be written as follows:

$$aG_A + G_B \geq bG_{LRS} \quad \text{(S14a)}$$

$$cG_A + G_B \geq \frac{b \times G_{LRS} \times w_{1B} + (-G_R - G_{IRS} + \alpha (G_{HRS} + G_R)) V_{SET}}{G_{LRS} \times w_{2B}} G_{LRS} \quad \text{(S14b)}$$

Similar to the former case, the intersection of the two decision boundaries is given to:

Intersection point (G_A, G_B) =

$$\left(-\frac{G_{IRS}+G_R+b\times G_{LRS}-\alpha(G_{HRS}+G_R+b\times G_{LRS})}{(-1+\alpha)(-1+\alpha)}, \frac{\alpha\left(G_{IRS}+G_R-\alpha(G_{HRS}+G_R)\right)-(-1+\alpha)b\times G_{LRS}}{(-1+\alpha)(-1+\alpha)V_{SET}}\right) \qquad \text{(S15)}$$

Substituting Equations (S13a), (S13b), and (S13c) into Equation (S15), and then the intersection point condition can be identical to (S12).

From the intersection point, one can draw 10 possible boundary lines that divide the input states. The potential gate can be defined by the combination of the two out of 10 possible boundary lines. Also, the sequence of input states being included in the boundary line can be different with respect to the intersection point as the line rotates in clockwise or counterclockwise direction around the intersection point, and it results in different gates. In this regard, the intersection trajectory line can be divided into 22 sections according to the sequence of the input states being included. Figure S7 shows the 11 representative intersection points that result in different input state classification sequence when $G_A > G_B$. At each point, the included input state sequence can be the follows:

- The included input states sequences as the boundary line rotates in clockwise direction from the horizontal line aroud the intersection (1)

Point 1: ($G_{A,0}$, $G_{B,0}$), ($G_{A,0}$, $G_{B,1}$), ($G_{A,1}$, $G_{B,0}$), ($G_{A,0}$, $G_{B,2}$), ($G_{A,1}$, $G_{B,1}$), ($G_{A,2}$, $G_{B,0}$), ($G_{A,1}$, $G_{B,2}$), ($G_{A,2}$, $G_{B,1}$) and ($G_{A,2}$, $G_{B,2}$)

Point 2: ($G_{A,0}$, $G_{B,0}$), ($G_{A,0}$, $G_{B,1}$), ($G_{A,1}$, $G_{B,0}$), ($G_{A,0}$, $G_{B,2}$), ($G_{A,1}$, $G_{B,1}$), ($G_{A,1}$, $G_{B,2}$), ($G_{A,2}$, $G_{B,0}$), ($G_{A,2}$, $G_{B,1}$) and ($G_{A,2}$, $G_{B,2}$)

Point 3: ($G_{A,0}$, $G_{B,0}$), ($G_{A,0}$, $G_{B,1}$), ($G_{A,0}$, $G_{B,2}$), ($G_{A,1}$, $G_{B,0}$), ($G_{A,1}$, $G_{B,1}$), ($G_{A,1}$, $G_{B,2}$), ($G_{A,2}$, $G_{B,0}$), ($G_{A,2}$, $G_{B,1}$) and ($G_{A,2}$, $G_{B,2}$)

Point 4: ($G_{A,0}$, $G_{B,0}$), ($G_{A,0}$, $G_{B,1}$), ($G_{A,0}$, $G_{B,2}$), ($G_{A,1}$, $G_{B,0}$), ($G_{A,1}$, $G_{B,1}$), ($G_{A,1}$, $G_{B,2}$), ($G_{A,2}$,

$G_{B,2}$), ($G_{A,2}$, $G_{B,1}$) and ($G_{A,2}$, $G_{B,0}$)

Point 5: ($G_{A,0}$, $G_{B,0}$), ($G_{A,0}$, $G_{B,1}$), ($G_{A,0}$, $G_{B,2}$), ($G_{A,1}$, $G_{B,2}$), ($G_{A,1}$, $G_{B,1}$), ($G_{A,1}$, $G_{B,0}$), ($G_{A,2}$, $G_{B,2}$), ($G_{A,2}$, $G_{B,1}$) and ($G_{A,2}$, $G_{B,0}$)

Point 6: ($G_{A,0}$, $G_{B,2}$), ($G_{A,0}$, $G_{B,1}$), ($G_{A,0}$, $G_{B,0}$), ($G_{A,1}$, $G_{B,2}$), ($G_{A,1}$, $G_{B,1}$), ($G_{A,1}$, $G_{B,0}$), ($G_{A,2}$, $G_{B,2}$), ($G_{A,2}$, $G_{B,1}$) and ($G_{A,2}$, $G_{B,0}$)

Point 7: ($G_{A,0}$, $G_{B,2}$), ($G_{A,0}$, $G_{B,1}$), ($G_{A,0}$, $G_{B,0}$), ($G_{A,1}$, $G_{B,2}$), ($G_{A,1}$, $G_{B,1}$), ($G_{A,2}$, $G_{B,2}$), ($G_{A,1}$, $G_{B,0}$), ($G_{A,2}$, $G_{B,1}$) and ($G_{A,2}$, $G_{B,0}$)

Point 8: ($G_{A,0}$, $G_{B,2}$), ($G_{A,0}$, $G_{B,1}$), ($G_{A,1}$, $G_{B,2}$), ($G_{A,0}$, $G_{B,0}$), ($G_{A,1}$, $G_{B,1}$), ($G_{A,2}$, $G_{B,2}$), ($G_{A,1}$, $G_{B,0}$), ($G_{A,2}$, $G_{B,1}$) and ($G_{A,2}$, $G_{B,0}$)

Point 9: ($G_{A,0}$, $G_{B,2}$), ($G_{A,0}$, $G_{B,1}$), ($G_{A,1}$, $G_{B,2}$), ($G_{A,0}$, $G_{B,0}$), ($G_{A,1}$, $G_{B,1}$), ($G_{A,2}$, $G_{B,2}$), ($G_{A,2}$, $G_{B,1}$), ($G_{A,1}$, $G_{B,0}$) and ($G_{A,2}$, $G_{B,0}$)

Point 10: ($G_{A,0}$, $G_{B,2}$), ($G_{A,0}$, $G_{B,1}$), ($G_{A,1}$, $G_{B,2}$), ($G_{A,0}$, $G_{B,0}$), ($G_{A,1}$, $G_{B,1}$), ($G_{A,1}$, $G_{B,0}$), ($G_{A,2}$, $G_{B,2}$), ($G_{A,2}$, $G_{B,1}$) and ($G_{A,2}$, $G_{B,0}$)

Point 11: ($G_{A,0}$, $G_{B,2}$), ($G_{A,1}$, $G_{B,2}$), ($G_{A,0}$, $G_{B,1}$), ($G_{A,0}$, $G_{B,0}$), ($G_{A,1}$, $G_{B,1}$), ($G_{A,2}$, $G_{B,2}$), ($G_{A,1}$, $G_{B,0}$), ($G_{A,2}$, $G_{B,1}$) and ($G_{A,2}$, $G_{B,0}$)

Similarly, Figure S8 shows another 11 cases when $G_B > G_A$.

- The included input states sequences as the boundary line rotates in counterclockwise direction from the vertical line around the intersection (2)

Point 1: ($G_{A,0}$, $G_{B,0}$), ($G_{A,1}$, $G_{B,0}$), ($G_{A,0}$, $G_{B,1}$), ($G_{A,2}$, $G_{B,0}$), ($G_{A,1}$, $G_{B,1}$), ($G_{A,0}$, $G_{B,2}$), ($G_{A,2}$, $G_{B,1}$),

($G_{A,1}$, $G_{B,2}$) and ($G_{A,2}$, $G_{B,2}$)

Point 2: ($G_{A,0}$, $G_{B,0}$), ($G_{A,1}$, $G_{B,0}$), ($G_{A,0}$, $G_{B,1}$), ($G_{A,2}$, $G_{B,0}$), ($G_{A,1}$, $G_{B,1}$), ($G_{A,2}$, $G_{B,1}$), ($G_{A,0}$, $G_{B,2}$), ($G_{A,1}$, $G_{B,2}$) and ($G_{A,2}$, $G_{B,2}$)

Point 3: ($G_{A,0}$, $G_{B,0}$), ($G_{A,1}$, $G_{B,0}$), ($G_{A,2}$, $G_{B,0}$), ($G_{A,0}$, $G_{B,1}$), ($G_{A,1}$, $G_{B,1}$), ($G_{A,2}$, $G_{B,1}$), ($G_{A,0}$,

$G_{B,2}$), ($G_{A,1}$, $G_{B,2}$) and ($G_{A,2}$, $G_{B,2}$)

Point 4: ($G_{A,0}$, $G_{B,0}$), ($G_{A,1}$, $G_{B,0}$), ($G_{A,2}$, $G_{B,0}$), ($G_{A,0}$, $G_{B,1}$), ($G_{A,1}$, $G_{B,1}$), ($G_{A,2}$, $G_{B,1}$), ($G_{A,2}$, $G_{B,2}$), ($G_{A,1}$, $G_{B,2}$) and ($G_{A,0}$, $G_{B,2}$)

Point 5: ($G_{A,0}$, $G_{B,0}$), ($G_{A,1}$, $G_{B,0}$), ($G_{A,2}$, $G_{B,0}$), ($G_{A,2}$, $G_{B,1}$), ($G_{A,1}$, $G_{B,1}$), ($G_{A,0}$, $G_{B,1}$), ($G_{A,2}$, $G_{B,2}$), ($G_{A,1}$, $G_{B,2}$) and ($G_{A,0}$, $G_{B,2}$)

Point 6: ($G_{A,2}$, $G_{B,0}$), ($G_{A,1}$, $G_{B,0}$), ($G_{A,0}$, $G_{B,0}$), ($G_{A,2}$, $G_{B,1}$), ($G_{A,1}$, $G_{B,1}$), ($G_{A,0}$, $G_{B,1}$), ($G_{A,2}$, $G_{B,2}$), ($G_{A,1}$, $G_{B,2}$) and ($G_{A,0}$, $G_{B,2}$)

Point 7: ($G_{A,2}$, $G_{B,0}$), ($G_{A,1}$, $G_{B,0}$), ($G_{A,0}$, $G_{B,0}$), ($G_{A,2}$, $G_{B,1}$), ($G_{A,1}$, $G_{B,1}$), ($G_{A,2}$, $G_{B,2}$), ($G_{A,0}$, $G_{B,1}$), ($G_{A,1}$, $G_{B,2}$) and ($G_{A,0}$, $G_{B,2}$)

Point 8: ($G_{A,2}$, $G_{B,0}$), ($G_{A,1}$, $G_{B,0}$), ($G_{A,2}$, $G_{B,1}$), ($G_{A,0}$, $G_{B,0}$), ($G_{A,1}$, $G_{B,1}$), ($G_{A,2}$, $G_{B,2}$), ($G_{A,0}$, $G_{B,1}$), ($G_{A,1}$, $G_{B,2}$) and ($G_{A,0}$, $G_{B,2}$)

Point 9: ($G_{A,2}$, $G_{B,0}$), ($G_{A,1}$, $G_{B,0}$), ($G_{A,2}$, $G_{B,1}$), ($G_{A,0}$, $G_{B,0}$), ($G_{A,1}$, $G_{B,1}$), ($G_{A,2}$, $G_{B,2}$), ($G_{A,1}$, $G_{B,2}$), ($G_{A,0}$, $G_{B,1}$) and ($G_{A,0}$, $G_{B,2}$)

Point 10: ($G_{A,2}$, $G_{B,0}$), ($G_{A,1}$, $G_{B,0}$), ($G_{A,2}$, $G_{B,1}$), ($G_{A,0}$, $G_{B,0}$), ($G_{A,1}$, $G_{B,1}$), ($G_{A,0}$, $G_{B,1}$), ($G_{A,2}$, $G_{B,2}$), ($G_{A,1}$, $G_{B,2}$) and ($G_{A,0}$, $G_{B,2}$)

Point 11: ($G_{A,2}$, $G_{B,0}$), ($G_{A,2}$, $G_{B,1}$), ($G_{A,1}$, $G_{B,0}$), ($G_{A,0}$, $G_{B,0}$), ($G_{A,1}$, $G_{B,1}$), ($G_{A,2}$, $G_{B,2}$), ($G_{A,0}$, $G_{B,1}$), ($G_{A,1}$, $G_{B,2}$) and ($G_{A,0}$, $G_{B,2}$)

The number of possible gates at each point is ${}_{10}H_2 = {}_{11}C_2$, 55, so the total number pf gate is 2×22×55, 2420. Considering duplicate gates and excluding the zero-input, one-input gates, and two-input gates when V_A=V_B, the exclusively possible two-input gates when $V_A \neq V_B$ is 484.

Putting all cases together, there are a total of 551 gates that can operate on a single clock. They are named potential ternary gates (PTG). The PTG are obtained only considering the output cell ignoring the state change of the input cells. The implementable gate should consider the input states, which is discussed in Supporting Note Ⅲ.

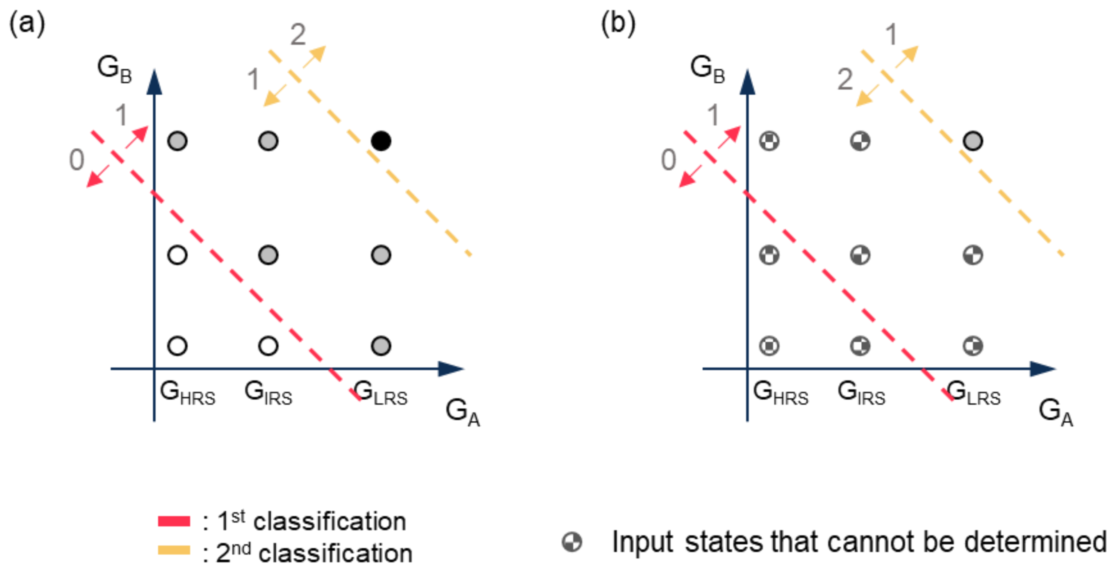

Figure S4 Acceptable and unacceptable example of decision boundaries selection regard to the switch sequence. a) Compliance and b) Violation of the first rule.

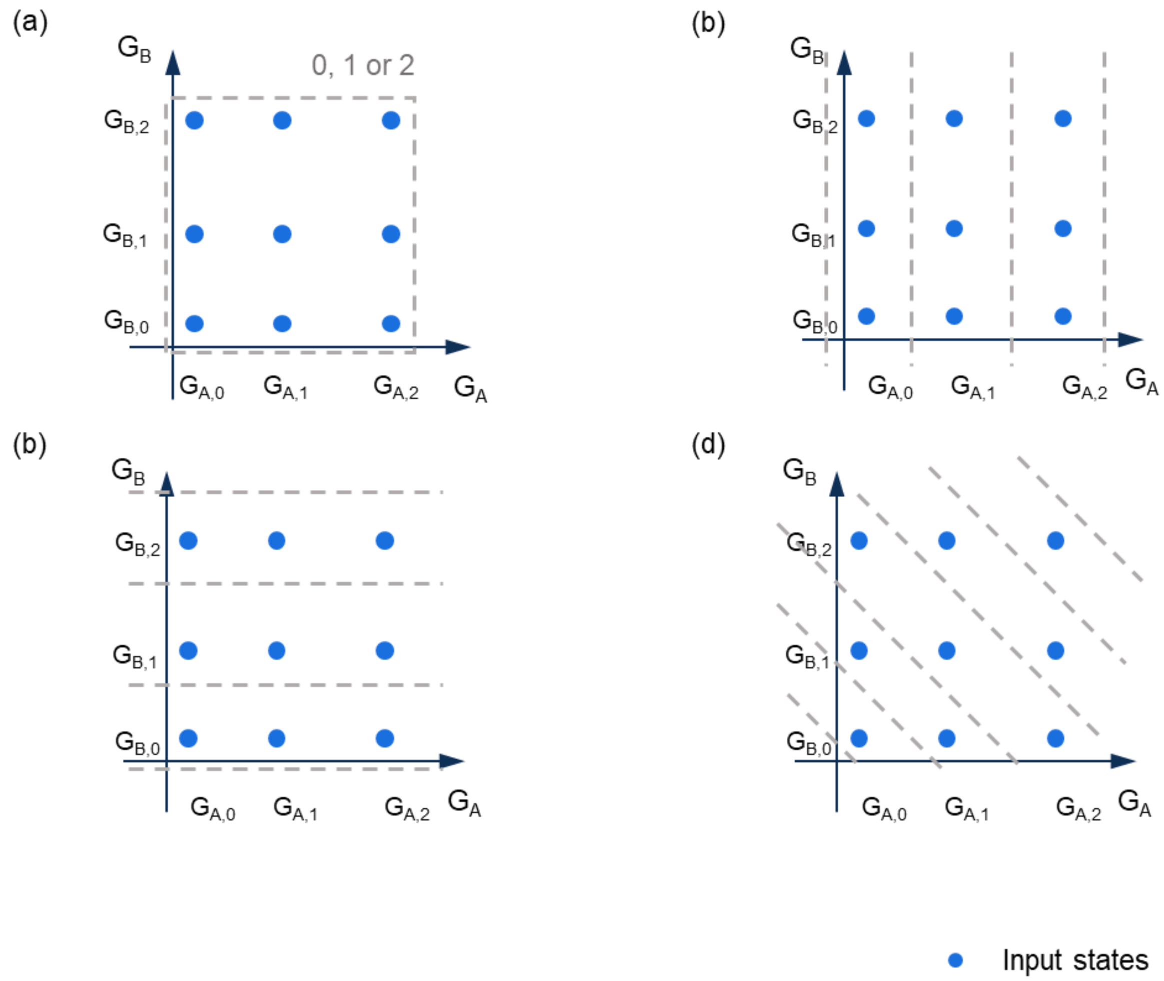

Figure S5 Possible classifications with the ternary stateful neural network (1). The dashed lines determine the order in which each input state is distinguished. The 1st and 2nd decision boundaries can choose one of them arbitrarily while obeying the first rule. a) Zero-input gates. b, c) One-input gates when input is G_A or $G_{B,}$ respectively. d) Two-input gates when the slope of both lines is -1.

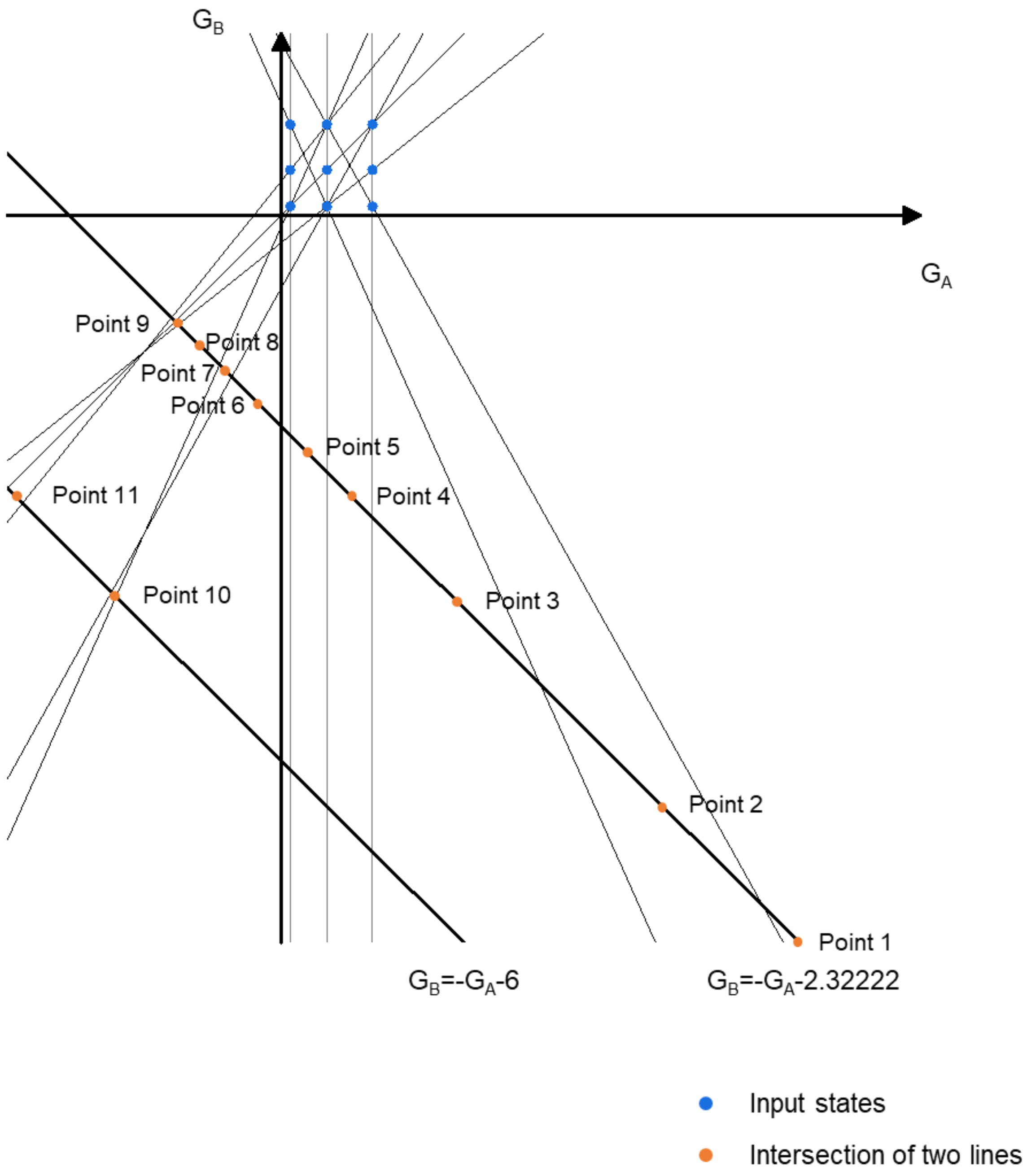

Figure S6 Possible classifications with the ternary stateful neural network (2). Two input gates when the intersection of two lines is on $\boldsymbol{G_B = -G_A - G_R + \frac{G_{IRS} - \alpha G_{HRS}}{(\alpha - 1)}}$. and x-coordinate of it is greater than y-coordinate. Substituting the characteristics of the developed ternary memristor, $\boldsymbol{\frac{G_{IRS} - \alpha G_{HRS}}{(\alpha - 1)}}$ is -2.3222. There are 11 points where input state points can be distinguished in a different order. Gray dashed lines as in Figure S6 are omitted.

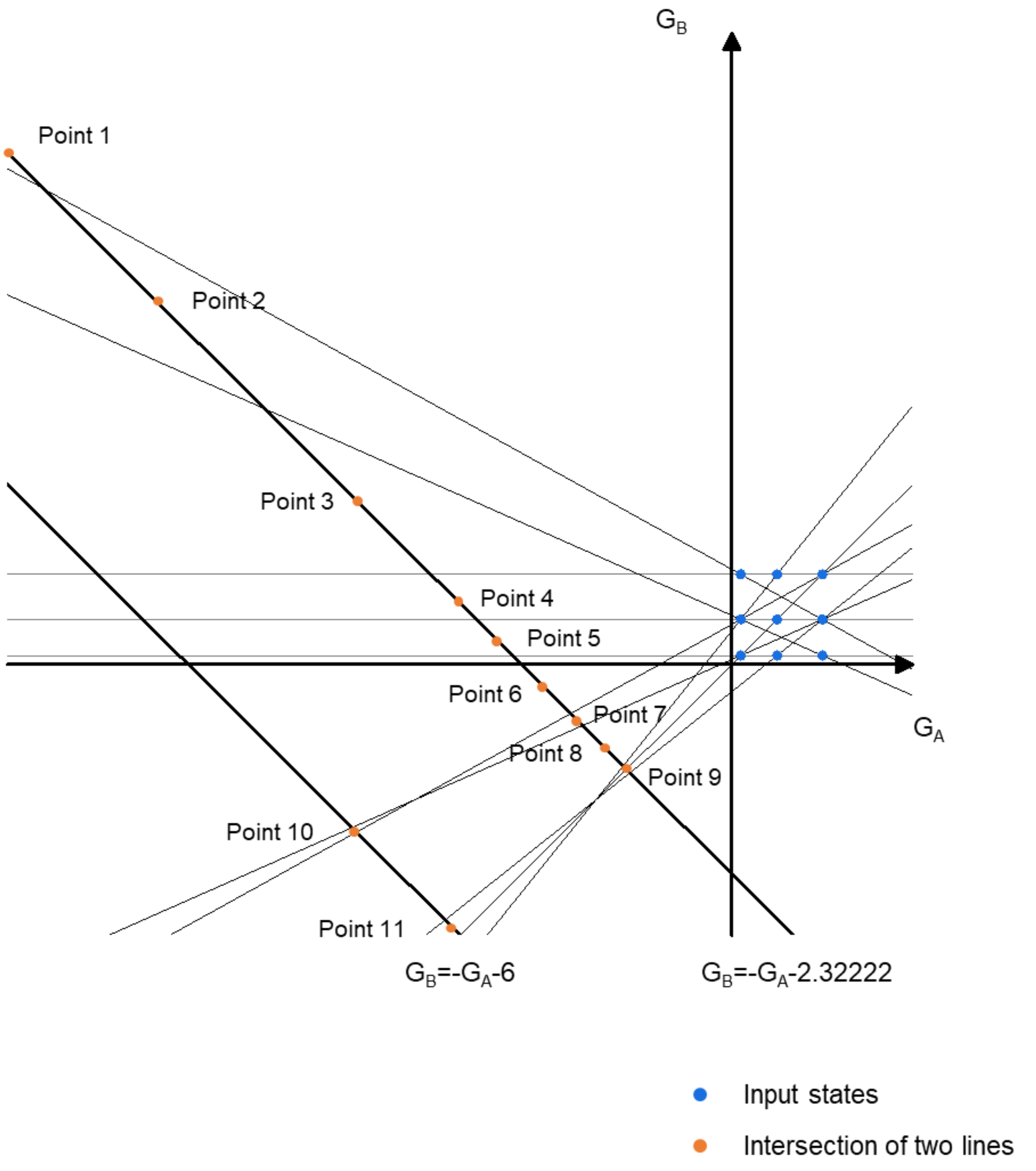

Figure S7 Possible classifications with the ternary stateful neural network (3). Two input gates when the intersection of two lines is on $\boldsymbol{G_B = -G_A - G_R + \frac{G_{IRS} - \alpha G_{HRS}}{(\alpha - 1)}}$ and y-coordinate of it is greater than x-coordinate. There are also 11 points where input states can be distinguished in a different order. Gray dashed lines as in Figure S6 are omitted.

Supporting Note Ⅲ.

A detailed methodology for investigating the ternary unit gates (TUGs) considering the input boundary lines

In the input cells, the required applied voltage conditions for keeping the original state are $v_M < \alpha V_{SET}$ for HRS cells, $V_{RESET(I\text{-}H)} < v_M < V_{SET}$ for IRS cells, and $V_{RESET(L\text{-}H)} < v_M$ for LRS cells, where v_M is the applied voltage across the memristor cell M. In implementing a particular gate, all input should obey the above conditions before changing the output, after partially changing the output, and after completely changing the output.

There are up to 81 input and output conditions that need to be considered with the given voltage condition. As an example, the voltage conditions that each cell complies with to implement a strong disjunction gate during the entire process are shown in Figure S9. The output cells to be switched to 1 should satisfy the red condition, and the output cells to be switched to 2 should satisfy the yellow condition. At the same time, the input cells should remain in their initial states. The set of voltage values for V_A, $V_{B,}$ and V_O used in Figure 2e is one of the solutions.

We solved all cases using Mathematica. In conclusion, we found that 157 gates are possible in the developed device, which are named as ternary unit gates (TUG). The truth tables for TUG are shown in Figure S10 and S11.

(a)

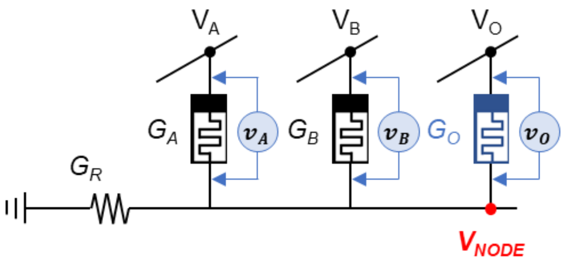

(b)

G_A	G_B	G_O
0	0	0
0	1	0
0	2	0
1	0	0
1	1	0
1	2	0
2	0	0
2	1	0
2	2	0

1st class. →

G_A	G_B	G_O’
0	0	0
0	1	1
0	2	1
1	0	1
1	1	1
1	2	1
2	0	1
2	1	1
2	2	1

2nd class. →

G_A	G_B	G_O”
0	0	0
0	1	1
0	2	2
1	0	1
1	1	2
1	2	2
2	0	2
2	1	2
2	2	2

(c)

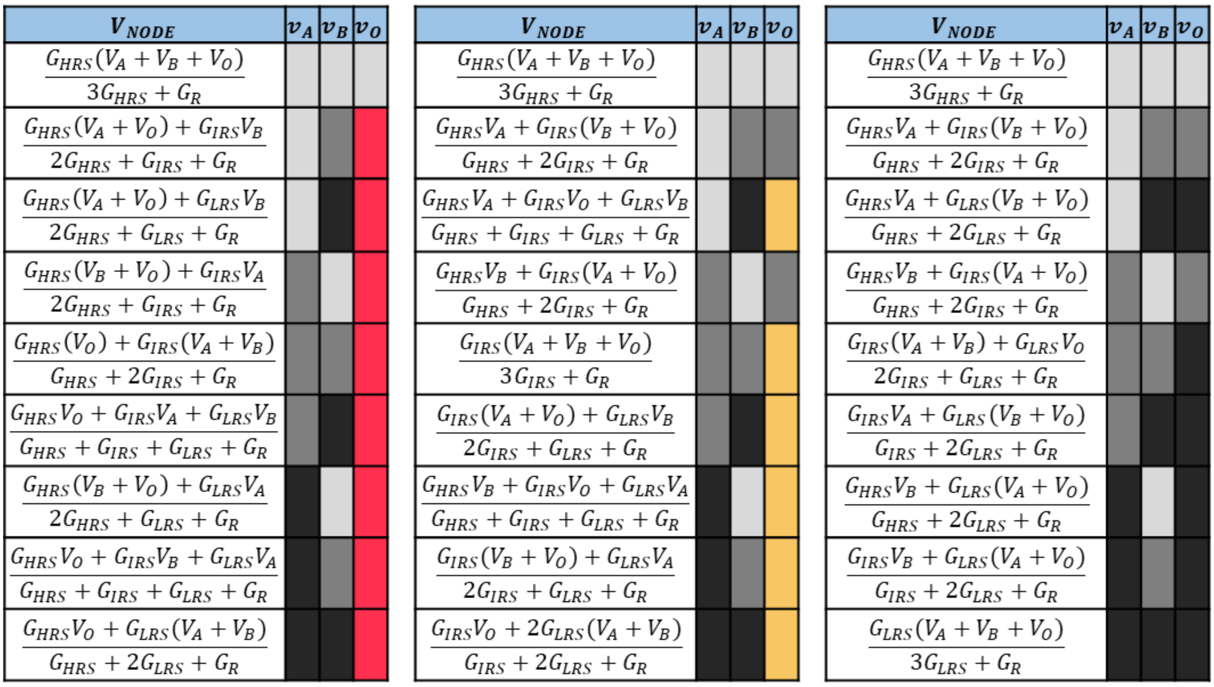

(d) $v_i = V_i - V_{NODE}$, where i equals A, B or O

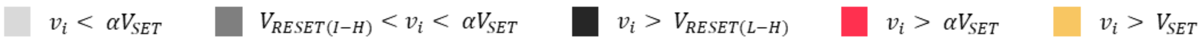

Figure S8 The voltage conditions for each cell to execute strong disjunction of Ł3. a) Circuit configuration for strong disjunction of Ł3. b) The truth tables for the state of each cell before and after the 1st classification and after the 2nd classification are listed. c) The V_{NODE} that changes according to input states and the voltage conditions for input and output cells are expressed as the color. d) Each color means a different voltage condition.

(a)

1	0	1	2
0	2	2	2
1	2	2	2
2	2	2	2

2	0	1	2
0	1	1	1
1	1	1	1
2	1	1	1

3	0	1	2
0	0	0	0
1	0	0	0
2	0	0	0

(b)

1	0	1	2
0	1	2	2
1	1	2	2
2	1	2	2

2	0	1	2
0	1	1	2
1	1	1	2
2	1	1	2

3	0	1	2
0	0	1	1
1	0	1	1
2	0	1	1

4	0	1	2
0	0	0	1
1	0	0	1
2	0	0	1

5	0	1	2
0	1	0	0
1	1	0	0
2	1	0	0

6	0	1	2
0	1	1	0
1	1	1	0
2	1	1	0

7	0	1	2
0	2	1	1
1	2	1	1
2	2	1	1

8	0	1	2
0	2	2	1
1	2	2	1
2	2	2	1

9	0	1	2
0	0	1	2
1	0	1	2
2	0	1	2

10	0	1	2
0	1	1	1
1	2	2	2
2	2	2	2

11	0	1	2
0	1	1	1
1	1	1	1
2	2	2	2

12	0	1	2
0	0	0	0
1	1	1	1
2	1	1	1

13	0	1	2
0	0	0	0
1	0	0	0
2	1	1	1

14	0	1	2
0	1	1	1
1	0	0	0
2	0	0	0

15	0	1	2
0	1	1	1
1	1	1	1
2	0	0	0

16	0	1	2
0	2	2	2
1	1	1	1
2	1	1	1

17	0	1	2
0	2	2	2
1	2	2	2
2	1	1	1

18	0	1	2
0	0	0	0
1	1	1	1
2	2	2	2

(c)

1	0	1	2
0	1	2	2
1	2	2	2
2	2	2	2

2	0	1	2
0	2	2	2
1	2	2	2
2	2	2	1

3	0	1	2
0	1	1	2
1	1	2	2
2	2	2	2

4	0	1	2
0	2	2	2
1	2	2	1
2	2	1	1

5	0	1	2
0	1	1	1
1	1	1	2
2	1	2	2

6	0	1	2
0	2	2	1
1	2	1	1
2	1	1	1

7	0	1	2
0	1	1	1
1	1	1	1
2	1	1	2

8	0	1	2
0	2	1	1
1	1	1	1
2	1	1	1

9	0	1	2
0	0	1	2
1	1	2	2
2	2	2	2

10	0	1	2
0	0	1	1
1	1	1	2
2	1	2	2

11	0	1	2
0	0	1	1
1	1	1	1
2	1	1	2

12	0	1	2
0	0	1	1
1	1	1	1
2	1	1	1

13	0	1	2
0	2	1	1
1	1	1	1
2	1	1	0

14	0	1	2
0	1	1	1
1	1	1	1
2	1	1	0

15	0	1	2
0	0	0	1
1	0	1	2
2	1	2	2

16	0	1	2
0	0	0	1
1	0	1	1
2	1	1	2

17	0	1	2
0	0	0	1
1	0	1	1
2	1	1	1

18	0	1	2
0	1	1	1
1	1	1	0
2	1	0	0

19	0	1	2
0	0	0	0
1	0	0	1
2	0	1	1

20	0	1	2
0	1	1	0
1	1	0	0
2	0	0	0

21	0	1	2
0	0	0	0
1	0	0	0
2	0	0	1

22	0	1	2
0	1	0	0
1	0	0	0
2	0	0	0

Figure S9 Truth tables for TUG (1). a) Zero-input, b) One-input, and c) Two-input when the slope of two boundaries is -1. The leftmost column means G_A, the first input, and the top row means G_B, the second input. The 15th gate of b) was used for the 1st clock of the INV gate implementation. The 9th gate of c) is strong disjunction gate.

1	0	1	2
0	2	2	2
1	2	2	2
2	1	2	2

2	0	1	2
0	2	2	1
1	2	2	2
2	2	2	2

3	0	1	2
0	2	2	2
1	1	2	2
2	1	2	2

4	0	1	2
0	2	2	2
1	2	2	2
2	1	1	2

5	0	1	2
0	2	1	1
1	2	2	2
2	2	2	2

6	0	1	2
0	2	2	1
1	2	2	1
2	2	2	2

7	0	1	2
0	1	2	2
1	1	2	2
2	1	1	2

8	0	1	2
0	1	1	1
1	2	2	1
2	2	2	2

9	0	1	2
0	2	2	2
1	1	2	2
2	1	1	1

10	0	1	2
0	2	1	1
1	2	2	1
2	2	2	1

11	0	1	2
0	1	2	2
1	1	1	2
2	1	1	2

12	0	1	2
0	1	1	1
1	2	1	1
2	2	2	2

13	0	1	2
0	2	2	2
1	1	1	2
2	1	1	1

14	0	1	2
0	2	1	1
1	2	1	1
2	2	2	1

15	0	1	2
0	1	1	2
1	1	1	2
2	1	1	1

16	0	1	2
0	1	2	2
1	1	1	1
2	1	1	1

17	0	1	2
0	1	1	1
1	1	1	1
2	2	2	1

18	0	1	2
0	1	1	1
1	2	1	1
2	2	1	1

19	0	1	2
0	1	1	2
1	1	1	1
2	1	1	1

20	0	1	2
0	1	1	1
1	1	1	1
2	2	1	1

21	0	1	2
0	1	1	2
1	1	1	2
2	0	1	1

22	0	1	2
0	1	1	2
1	1	1	1
2	0	1	1

23	0	1	2
0	1	1	1
1	1	1	1
2	0	1	1

24	0	1	2
0	1	1	0
1	1	1	1
2	2	2	1

25	0	1	2
0	1	1	0
1	1	1	1
2	2	1	1

26	0	1	2
0	1	1	0
1	1	1	1
2	1	1	1

27	0	1	2
0	1	1	2
1	0	1	2
2	0	1	1

28	0	1	2
0	1	1	2
1	0	1	1
2	0	1	1

29	0	1	2
0	1	1	1
1	0	1	1
2	0	1	1

30	0	1	2
0	1	1	1
1	1	1	1
2	0	0	1

31	0	1	2
0	1	0	0
1	1	1	1
2	2	2	1

32	0	1	2
0	1	0	0
1	1	1	1
2	2	1	1

33	0	1	2
0	1	0	0
1	1	1	1
2	1	1	1

34	0	1	2
0	1	1	0
1	1	1	0
2	1	1	1

35	0	1	2
0	0	1	2
1	0	1	2
2	0	1	1

36	0	1	2
0	0	1	2
1	0	1	1
2	0	1	1

37	0	1	2
0	0	0	0
1	1	1	1
2	2	2	1

38	0	1	2
0	0	0	0
1	1	1	1
2	2	1	1

39	0	1	2
0	0	1	2
1	0	1	1
2	0	0	1

40	0	1	2
0	0	1	1
1	0	1	1
2	0	0	1

41	0	1	2
0	0	0	0
1	1	1	0
2	2	1	1

42	0	1	2
0	0	0	0
1	1	1	0
2	1	1	1

43	0	1	2
0	1	1	1
1	0	1	1
2	0	0	0

44	0	1	2
0	1	0	0
1	1	1	0
2	1	1	0

45	0	1	2
0	0	1	1
1	0	0	1
2	0	0	1

46	0	1	2
0	0	0	0
1	1	0	0
2	1	1	1

47	0	1	2
0	1	1	1
1	0	0	1
2	0	0	0

48	0	1	2
0	1	0	0
1	1	0	0
2	1	1	0

49	0	1	2
0	0	0	1
1	0	0	1
2	0	0	0

50	0	1	2
0	0	1	1
1	0	0	0
2	0	0	0

51	0	1	2
0	0	0	0
1	0	0	0
2	1	1	0

52	0	1	2
0	0	0	0
1	1	0	0
2	1	0	0

53	0	1	2
0	0	0	1
1	0	0	0
2	0	0	0

54	0	1	2
0	0	0	0
1	0	0	0
2	1	0	0

55	0	1	2
0	1	2	2
1	1	2	2
2	2	2	2

56	0	1	2
0	1	1	2
1	2	2	2
2	2	2	2

57	0	1	2
0	2	2	2
1	2	2	2
2	2	1	1

58	0	1	2
0	2	2	2
1	2	2	1
2	2	2	1

59	0	1	2
0	2	2	2
1	1	2	2
2	1	1	2

60	0	1	2
0	2	1	1
1	2	2	1
2	2	2	2

61	0	1	2
0	1	1	2
1	1	2	2
2	1	2	2

62	0	1	2
0	1	1	1
1	1	2	2
2	2	2	2

63	0	1	2
0	2	2	2
1	2	2	1
2	1	1	1

64	0	1	2
0	2	2	1
1	2	2	1
2	2	1	1

65	0	1	2
0	1	1	2
1	1	1	2
2	1	2	2

66	0	1	2
0	1	1	1
1	1	1	2
2	2	2	2

67	0	1	2
0	2	2	2
1	2	1	1
2	1	1	1

68	0	1	2
0	2	2	1
1	2	1	1
2	2	1	1

69	0	1	2
0	1	2	2
1	1	1	2
2	1	1	1

70	0	1	2
0	1	1	1
1	2	1	1
2	2	2	1

71	0	1	2
0	1	1	1
1	1	1	2
2	1	1	2

72	0	1	2
0	1	1	1
1	1	1	1
2	1	2	2

73	0	1	2
0	2	2	1
1	1	1	1
2	1	1	1

74	0	1	2
0	2	1	1
1	2	1	1
2	1	1	1

75	0	1	2
0	0	1	2
1	1	1	2
2	1	2	2

76	0	1	2
0	0	1	1
1	1	1	2
2	2	2	2

77	0	1	2
0	0	1	2
1	1	1	2
2	1	1	2

78	0	1	2
0	0	1	1
1	1	1	1
2	2	2	2

79	0	1	2
0	0	1	1
1	1	1	2
2	1	1	2

80	0	1	2
0	0	1	2
1	1	1	2
2	1	1	1

81	0	1	2
0	0	1	1
1	1	1	1
2	1	2	2

82	0	1	2
0	0	1	1
1	1	1	1
2	2	2	1

83	0	1	2
0	0	1	2
1	1	1	1
2	1	1	1

84	0	1	2
0	0	1	1
1	1	1	1
2	2	1	1

85	0	1	2
0	0	1	2
1	0	1	2
2	1	1	1

86	0	1	2
0	0	1	1
1	0	1	1
2	1	1	2

87	0	1	2
0	0	1	2
1	0	1	1
2	1	1	1

88	0	1	2
0	0	1	1
1	0	1	1
2	1	1	1

89	0	1	2
0	0	0	1
1	1	1	1
2	2	2	1

90	0	1	2
0	0	0	1
1	1	1	1
2	1	1	2

91	0	1	2
0	0	0	1
1	1	1	1
2	2	1	1

92	0	1	2
0	0	0	1
1	1	1	1
2	1	1	1

93	0	1	2
0	1	1	1
1	1	1	1
2	1	0	0

94	0	1	2
0	1	1	1
1	1	1	0
2	1	1	0

95	0	1	2
0	0	0	1
1	0	1	2
2	1	1	2

96	0	1	2
0	0	0	1
1	0	1	1
2	1	2	2

97	0	1	2
0	1	1	2
1	0	1	1
2	0	0	1

98	0	1	2
0	1	1	1
1	0	1	1
2	0	0	1

99	0	1	2
0	1	0	0
1	1	1	0
2	2	1	1

100	0	1	2
0	1	0	0
1	1	1	0
2	1	1	1

101	0	1	2
0	0	0	1
1	0	1	1
2	0	1	1

102	0	1	2
0	0	0	0
1	0	1	1
2	1	1	1

103	0	1	2
0	1	1	1
1	1	1	0
2	0	0	0

104	0	1	2
0	1	1	0
1	1	1	0
2	1	0	0

105	0	1	2
0	0	0	1
1	0	0	1
2	0	1	1

106	0	1	2
0	0	0	0
1	0	0	1
2	1	1	1

107	0	1	2
0	1	1	1
1	1	0	0
2	0	0	0

108	0	1	2
0	1	1	0
1	1	0	0
2	1	0	0

109	0	1	2
0	0	1	1
1	0	0	1
2	0	0	0

110	0	1	2
0	0	0	0
1	1	0	0
2	1	1	0

111	0	1	2
0	0	0	0
1	0	0	1
2	0	0	1

112	0	1	2
0	0	0	0
1	0	0	0
2	0	1	1

113	0	1	2
0	1	1	0
1	0	0	0
2	0	0	0

114	0	1	2
0	1	0	0
1	1	0	0
2	0	0	0

Figure S10 Truth tables for TUG (2). Two-input gates when the intersection of two linear lines is above $G_B = -G_A-G_R-2.32222$. The leftmost column means G_A, the first input, and the top row means G_B, the second input. The 23rd gate was used for the 1st clock of the IMP gate implementation.

(a) Truth table of *T*()

Input (A)	Output (O)
0	1
1	1
2	1

(b) In stateful logic,

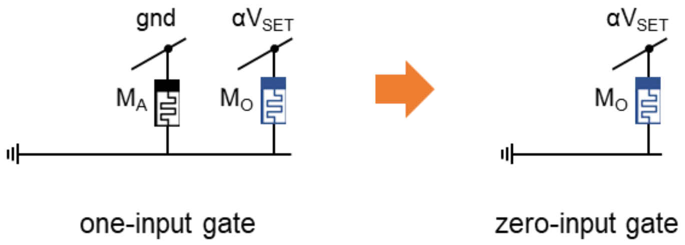

(c) Demonstration of **0**, **1**, and *T*()

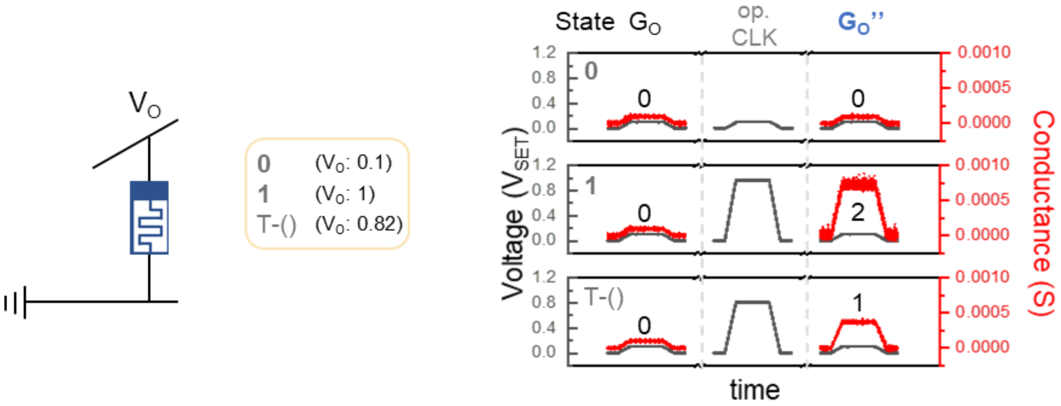

Figure S11 Conversion *T*() into zero-input gate in stateful logic and experimental demonstration of zero-input gates. a) Truth table of *T*(). b) In stateful logic, *T*() can be implemented using only output cell. c) Operation of **0**, **1**, and *T*() gates.

(a) Truth table of ternary full adder

Input			Output	
			C	S
0	0	0	0	0
0	0	1	0	1
0	0	2	0	2
0	1	0	0	1
0	1	1	0	2
0	1	2	1	0
0	2	0	0	2
0	2	1	1	0
0	2	2	1	1
1	0	0	0	1
1	0	1	0	2
1	0	2	1	0
1	1	0	0	2
1	1	1	1	0
1	1	2	1	1
1	2	0	1	0
1	2	1	1	1
1	2	2	1	2
2	0	0	1	2
2	0	1	0	0
2	0	2	1	1
2	1	0	1	0
2	1	1	1	1
2	1	2	1	2
2	2	0	1	1
2	2	1	1	2
2	2	2	2	2

case: 27

(b) Simplifed truth table of ternary full adder

Input			Output			
			C	S	S1	S2
0	0	0	0	0	0	0
0	0	1	0	1	0	1
0	0	2	0	2	1	1
0	1	2	1	0	0	0
0	2	2	1	1	0	1
1	2	2	1	2	1	1
2	2	2	2	0	0	0

case: 7

Figure S12 Two types of truth tables of ternary full adder. a) Original truth table. b) Simplified truth table. The total number of cases is reduced from 27 to 7.

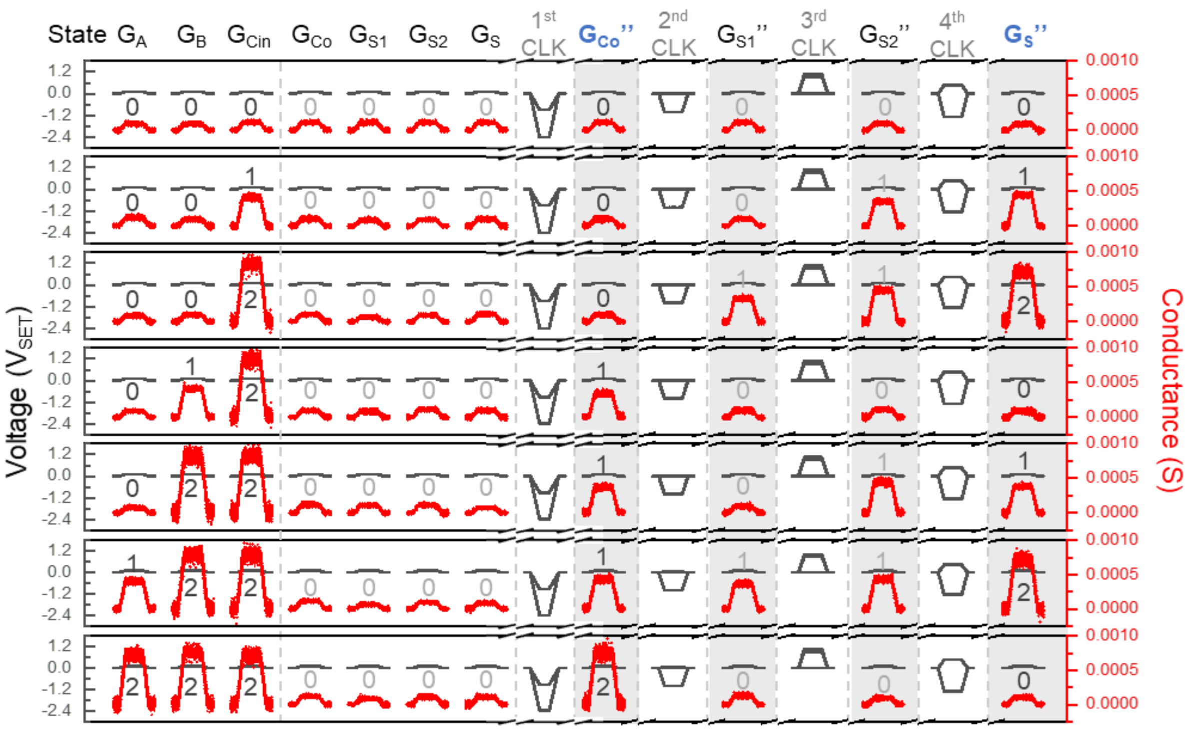

Figure S13 Experimental demonstration of ternary full adder. Carry-out operation needs one clock. After 1st clock, the carry-out state is used as an input for computing S1 and S2 in 2nd and 3rd clock. In the final clock, Sum operation was done using strong disjunction of S1 and S2.

In extra clock,

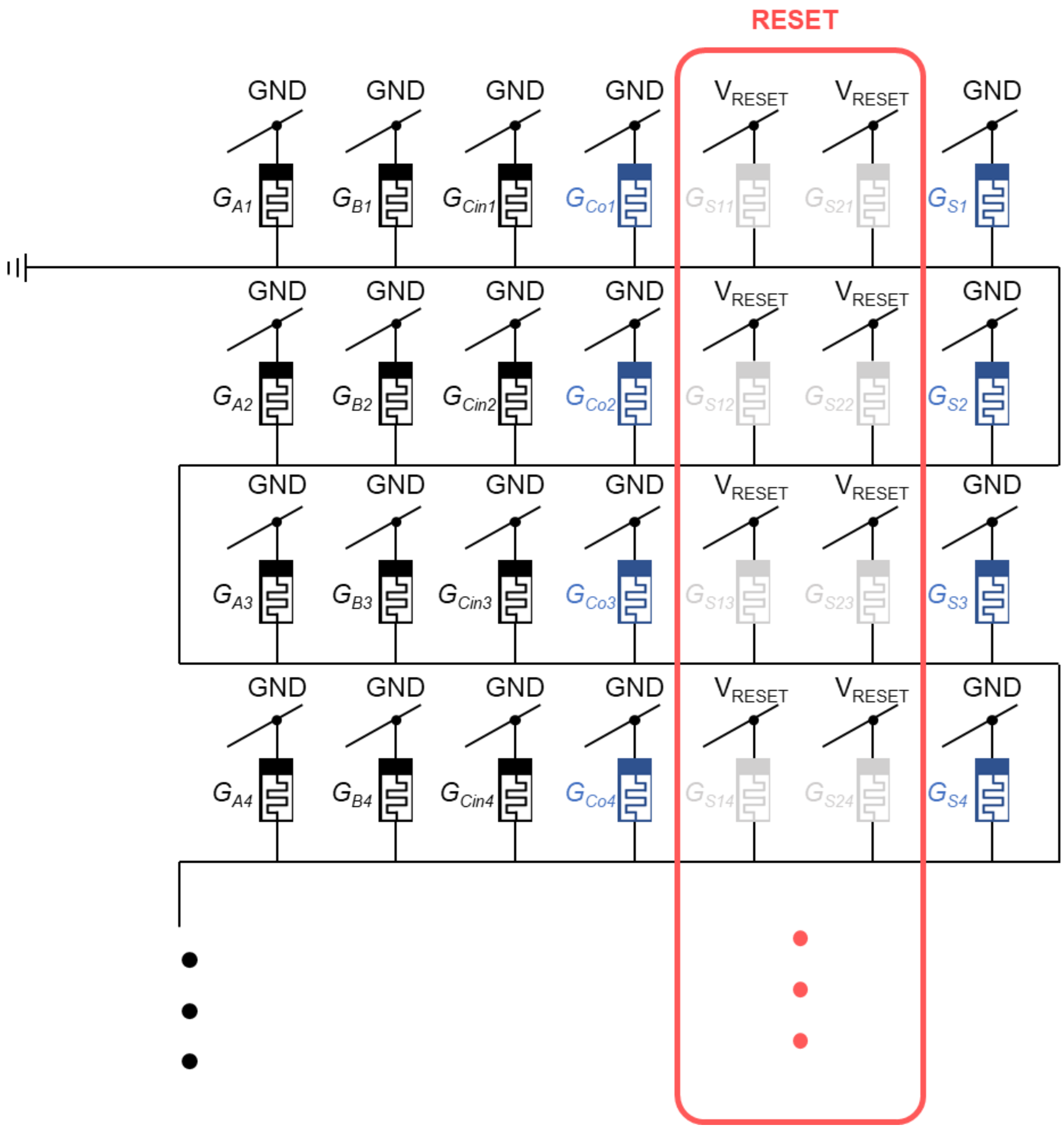

Figure S14 Extra clock to reset the unnecessary cells after computation. It effectively reduces required cells for ternary adder.

References

[1] K. M. Kim, S. R. Lee, S. Kim, M. Chang, C. S. Hwang, *Adv. Funct. Mater.* **2015**, *25*, 1527.

[2] K. M. Kim, C. S. Hwang, *Appl. Phys. Lett.* **2009**, *94*, 1.

[3] K. M. Kim, B. J. Choi, Y. C. Shin, S. Choi, C. S. Hwang, *Appl. Phys. Lett.* **2007**, *91*, 7.

[4] J. P. Strachan, A. C. Torrezan, F. Miao, M. D. Pickett, J. Joshua Yang, W. Yi, G. Medeiros-Ribeiro, R. Stanley Williams, *IEEE Trans. Electron Devices* **2013**, *60*, 2194.
